# Bidirectional modulation of TCA cycle metabolites and anaplerosis by metformin and its combination with SGLT2i

**DOI:** 10.1186/s12933-024-02288-x

**Published:** 2024-06-12

**Authors:** Makoto Harada, Jonathan Adam, Marcela Covic, Jianhong Ge, Stefan Brandmaier, Caroline Muschet, Jialing Huang, Siyu Han, Martina Rommel, Markus Rotter, Margit Heier, Robert P. Mohney, Jan Krumsiek, Gabi Kastenmüller, Wolfgang Rathmann, Zhongmei Zou, Sven Zukunft, Markus F. Scheerer, Susanne Neschen, Jerzy Adamski, Christian Gieger, Annette Peters, Donna P. Ankerst, Thomas Meitinger, Tanya L. Alderete, Martin Hrabe de Angelis, Karsten Suhre, Rui Wang-Sattler

**Affiliations:** 1 Institute of Translational Genomics, Helmholtz Zentrum München, German Research Center for Environmental Health, Neuherberg, Germany; 2 German Center for Diabetes Research (DZD), Neuherberg, Germany; 3 Institute of Epidemiology, Helmholtz Zentrum München, German Research Center for Environmental Health, Neuherberg, Germany; 4 Research Unit of Molecular Epidemiology, Institute of Epidemiology, Helmholtz Zentrum München, German Research Center for Environmental Health, Neuherberg, Germany; 5 TUM School of Medicine and Health, Technical University of Munich, Munich, Germany; 6 Institute of Experimental Genetics, Helmholtz Zentrum München, German Research Center for Environmental Health, Neuherberg, Germany; 7 KORA Study Centre, University Hospital of Augsburg, Augsburg, Germany; 8 Metabolon, Inc., Durham, NC USA; 9 Institute of Computational Biology, Helmholtz Zentrum München, German Research Center for Environmental Health, Neuherberg, Germany; 10 Institute for Biometrics and Epidemiology, German Diabetes Center, Leibniz Center for Diabetes Research at Heinrich Heine University, Düsseldorf, Germany; 11 Institute of Medicinal Plant Development, Chinese Academy of Medical Sciences and Peking Union Medical College, Beijing, China; 12 Department of Biochemistry, Yong Loo Lin School of Medicine, National University of Singapore, Singapore, Singapore; 13 Institute of Biochemistry, Faculty of Medicine, University of Ljubljana, Ljubljana, Slovenia; 14 Institute for Medical Information Processing, Biometry, and Epidemiology, Pettenkofer School of Public Health, Ludwig Maximilian University of Munich (LMU), Munich, Germany; 15 Departments of Mathematics and Life Science Systems, Technical University of Munich (TUM), Garching, Germany; 16 Institute of Human Genetics, Klinikum Rechts der Isar, TUM, Munich, Germany; 17 Department of Integrative Physiology, University of Colorado Boulder, Boulder, USA; 18 Chair of Experimental Genetics, TUM School of Life Sciences, TUM, Freising, Germany; 19 Department of Physiology and Biophysics, Weill Cornell Medicine - Qatar, Education City - Qatar Foundation, Doha, Qatar

**Keywords:** Pharmacometabolomics, Metformin, SGLT2 inhibitors, TCA cycle, Anaplerosis, Anti-inflammatory effects, Metabolic dysfunction-associated steatotic liver disease (MASLD), Type 2 diabetes

## Abstract

**Background:**

Metformin and sodium-glucose-cotransporter-2 inhibitors (SGLT2i) are cornerstone therapies for managing hyperglycemia in diabetes. However, their detailed impacts on metabolic processes, particularly within the citric acid (TCA) cycle and its anaplerotic pathways, remain unclear. This study investigates the tissue-specific metabolic effects of metformin, both as a monotherapy and in combination with SGLT2i, on the TCA cycle and associated anaplerotic reactions in both mice and humans.

**Methods:**

Metformin-specific metabolic changes were initially identified by comparing metformin-treated diabetic mice (MET) with vehicle-treated db/db mice (VG). These findings were then assessed in two human cohorts (KORA and QBB) and a longitudinal KORA study of metformin-naïve patients with Type 2 Diabetes (T2D). We also compared MET with db/db mice on combination therapy (SGLT2i + MET). Metabolic profiling analyzed 716 metabolites from plasma, liver, and kidney tissues post-treatment, using linear regression and Bonferroni correction for statistical analysis, complemented by pathway analyses to explore the pathophysiological implications.

**Results:**

Metformin monotherapy significantly upregulated TCA cycle intermediates such as malate, fumarate, and α-ketoglutarate (α-KG) in plasma, and anaplerotic substrates including hepatic glutamate and renal 2-hydroxyglutarate (2-HG) in diabetic mice. Downregulated hepatic taurine was also observed. The addition of SGLT2i, however, reversed these effects, such as downregulating circulating malate and α-KG, and hepatic glutamate and renal 2-HG, but upregulated hepatic taurine. In human T2D patients on metformin therapy, significant systemic alterations in metabolites were observed, including increased malate but decreased citrulline. The bidirectional modulation of TCA cycle intermediates in mice influenced key anaplerotic pathways linked to glutaminolysis, tumorigenesis, immune regulation, and antioxidative responses.

**Conclusion:**

This study elucidates the specific metabolic consequences of metformin and SGLT2i on the TCA cycle, reflecting potential impacts on the immune system. Metformin shows promise for its anti-inflammatory properties, while the addition of SGLT2i may provide liver protection in conditions like metabolic dysfunction-associated steatotic liver disease (MASLD). These observations underscore the importance of personalized treatment strategies.

**Supplementary Information:**

The online version contains supplementary material available at 10.1186/s12933-024-02288-x.

## Background

Metformin is widely recognized as a primary treatment option for non-insulin-dependent type 2 diabetes (T2D) [1]. Beyond its established benefits in blood sugar control and weight management, recent research has unveiled the pleiotropic effects of metformin, including potential anti-cancer effects, such as a decreased proliferation of cancer cells [2, 3], cardiovascular benefits such as lowered low-density lipoprotein cholesterol [4], and reduced inflammation and fibrosis [5, 6]. However, it is important to acknowledge that metformin is not without potential side effects, including gastrointestinal issues and, in rare cases, lactic acidosis [7].

Clinical guidelines now recommend the use of sodium-glucose-cotransporter-2 inhibitors (SGLT2i) as add-on therapy with metformin to enhance glycemic control [8, 9]. However, the effects of metformin monotherapy and its combination with SGLT2i on tissue-specific metabolism, particularly the citric acid, also known as tricarboxylic acid (TCA) cycle and anaplerosis in the liver and kidneys, have not been extensively investigated.

Both the liver and kidneys are integral to metformin's pharmacokinetics: the liver primarily processes the drug post-absorption, and the kidneys subsequently handle its clearance [10]. SGLT2 inhibitors, on the other hand, impede renal glucose reabsorption, promoting its urinary excretion [11]. Appreciating the tissue-specific actions of metformin and its interactions with SGLT2i is essential to refine treatment modalities for T2D and to fully understand the metabolic shifts induced by these drugs.

Pharmacometabolomics studies in animals and humans have provided further insights into the comprehensive effects of metformin, highlighting its significant influence on nitric oxide (NO) and urea cycles [12–14]. Non-targeted metabolomics has played a pivotal role in the research and development of biomarkers and screening assays for T2D [15–17]. Previous studies utilizing this approach have observed significantly lower citrulline levels in diabetic and non-diabetic individuals treated with metformin, as well as in peripheral tissues of metformin-treated diabetic mice [13, 14]. These findings suggest that metformin treatment alters urea and NO production by activating the endothelial NO synthase (eNOS) and NO biosynthesis, which could mediate its benefits in reducing cardiovascular sequels of T2D [18].

This non-targeted metabolomics study aims to examine the tissue-specific effects of metformin, both as a monotherapy and in combination with SGLT2i, on the plasma, hepatic and renal metabolite profiles in obese diabetic mice. We seek to corroborate our findings through the analysis of serum and plasma from T2D patients undergoing metformin treatment in the longitudinal KORA (Cooperative Health Research in the Region of Augsburg) study and the cross-sectional QBB study (Qatar Biobank). Our research endeavors to offer clinically relevant insights that could pave the way for personalized diabetes management approaches.

## Methods

### Study design

We firstly assessed the metabolic alterations in diabetic (db/db) mice treated with metformin (MET) compared to vehicle-gavaged db/db mice (VG), focusing on the analysis of significant tissue-specific metabolite differences (plasma, liver, kidney) between the MET and VG groups. Additionally, we evaluated whether the metabolic changes attributed to metformin were independent of leptin receptor (Lepr) deficiency by comparing the VG group to wild type (WT) mice.

We then examined the metformin-associated metabolites, initially identified in murine plasma, in two human cross-sectional studies: KORA and QBB. In these studies, we compared metformin-treated type 2 diabetes patients (mt-T2D) with T2D patients who were not treated with antidiabetic drugs (ndt-T2D). Metabolites replicated in these cross-sectional cohorts were further validated in a longitudinal KORA study, focusing on metformin-naïve participants at baseline.

Lastly, we compared the metabolic profiles of db/db mice treated with combination therapy (SGLT2i + MET) versus those on metformin monotherapy (MET) across the three tissues. For each of these comparisons, we performed linear regression analysis for pairwise comparisons. We also conducted tissue-specific pathway analyses to elucidate the biological relevance of the identified metabolites, thereby enhancing our understanding of the metabolic effects of these antidiabetic therapies.

### Metformin and SGLT2i intervention study

Pharmacological studies were conducted in compliance with FELASA protocols using 40 male mice including 10 wild-type (WT) and 30 db/db (BKS.Cg Dock7m + / + Leprdb/J) mice. Starting from 3 weeks of age, all db/db mice were fed a high-fat diet (HFD) (S0372 E010, ssniff Spezialdiäten, Soest, Germany) [19]. After 3 weeks of being on the HFD, the 6-week-old animals were treated for 2 weeks via gavage once a day between 5:00 and 6:00 p.m. before the onset of the dark phase (6:00 p.m.). The treatment groups consisted of 10 vehicle-gavaged (VG) diabetic mice that were vehicle-gavaged with a solution of 5% solutol and 95% hydroxyethylcellulose, 10 MET mice treated with 300 mg/kg metformin (Sigma Aldrich, Taufkirchen, Germany), and 10 SGLT2i + MET mice treated with 30 mg/kg SGLT2i (AVE2268, Sanofi AG, Frankfurt, Germany) and 300 mg/kg metformin (Sigma Aldrich, Taufkirchen, Germany) [20].

After the completion of the treatment period, which lasted for 2 weeks, the 8-week-old mice (± 3 days) underwent a fasting period of four hours in the afternoon, with sample collection (sacrifice) occurring between 17:00 and 18:00, four hours post-treatment. An overdose of isoflurane was used for euthanasia, and immediate blood and organ collection were performed as previously reported [19, 21]. All available blood was gathered from the vena cava into a 1.5 ml tube and centrifuged at 10,000 × g for 2 min at 4 °C to separate the plasma, and tissues were freeze-clamped. All samples were stored at – 80 °C until further analyses.

### KORA and QBB human studies

KORA is a population-based cohort conducted in southern Germany [22]. The baseline survey, Survey 4 (S4), included 4,261 individuals examined between 1999 and 2001. The follow-up survey (F4) occurred from 2006 to 2008, involved 3,080 individuals [23]. For this analysis, only participants with non-targeted metabolite profiles were included. Those who did not undergo overnight fasting, had Type 1 diabetes or drug-induced diabetes, were treated with insulin or both insulin and metformin, or took glucose-lowering oral medication other than metformin were excluded. In the cross-sectional F4 investigation, patients with T2D were included. The current study examined 184 T2D patients, including 70 individuals undergoing metformin therapy (mt-T2D). It should be noted that many individuals in the 114 ndt-T2D patients were on other medications, including those for lipid management. For the longitudinal analysis from KORA S4 to F4, participants who were naïve to metformin at baseline S4 and had non-targeted metabolite profiles at both the S4 and F4 surveys were included.

QBB is a population-based study conducted in Qatar, established in 2012 [24, 25]. The QBB initiative aims to provide a rich research platform reflecting the unique genetic and environmental background of the Qatari population. The biobank supports advanced research into the causes and mechanisms of diseases and health patterns within the local context. For this study, data from the QBB pilot study was utilized, which recruited 1209 participants between December 2012 and February 2014. Participants include Qatari nationals and long-term residents living in Qatar for 15 years or more, aged 18 and above. Similar to the KORA F4 study, patients with T2D were included. The QBB study comprised a sample set of 294 individuals, including 146 with mt-T2D.

### Non-targeted metabolite profiling using the metabolon analytical system

Non-targeted metabolite profiling was performed using samples from various murine tissues, KORA serum, and QBB plasma, using the Metabolon analytical system (Metabolon Inc., Durham, North Carolina, USA).

For human samples, the KORA F4 study was processed in 2009 with the HD2 platform. The KORA S4 samples were subsequently analyzed in 2011 using the same HD2 platform, and the QBB study samples underwent analysis in 2019 with the upgraded HD4 platform. Concurrently, murine samples from the Mouse200 project were processed in 2011, which included plasma, liver, and kidney tissues, also utilizing the HD2 platform.

The Metabolon system employs a non-targeted approach, utilizing semi-quantitative liquid chromatography tandem mass spectrometry (LC–MS/MS) and gas chromatography mass spectrometry (GC–MS) to identify a wide spectrum of metabolites, including both named structures and unidentified compounds [17]. For an in-depth methodology, please refer to the supplementary information provided in Additional File 2.

Quality control (QC) protocols were consistently applied across all samples as delineated in the literature [13, 26]. Metabolites with more than 20% missing values for any given tissue type were excluded. Similarly, any samples or running days containing more than 10% missing values for metabolites were also omitted from the study. Following QC, relative ion counts were normalized per tissue type for each run day and subsequently natural log-transformed to stabilize variance. The missing values were then imputed with Multivariate Imputation by Chained Equations (MICE) [27].

From these metabolites, 136 were common across the three murine tissues, with approximately 86% of these being structurally identified. In addition, we identified 118 plasma-specific, 119 kidney-specific, and 132 liver-specific metabolites, as itemized in Supplementary Table 1 (Additional File 1). These findings are detailed in Supplementary Table 1, within Additional File 1. Post-QC analysis revealed a total of 716 metabolites, which included 351 plasma metabolites, 391 liver metabolites, and 447 kidney metabolites (Supplementary Table 1, Additional File 1). From these metabolites, 136 were common across the three murine tissues, with approximately 86% of these being structurally identified. we identified 118 plasma-specific, 119 kidney-specific, and 132 liver-specific metabolites, as itemized in Supplementary Table 1 (Additional File 1). In addition, we identified 118 plasma-specific, 119 kidney-specific, and 132 liver-specific metabolites (Supplementary Table 1, Additional File 1).

### Statistical analysis

In the mouse study, three pairwise comparisons were conducted to assess the effects of mono- and combination therapy in diabetic mice (MET versus VG and SGLT2i + MET versus MET) and the impact of the diabetes-prone genetic background (VG versus WT mice). Linear regression analysis was used, with the relative metabolite concentration as the outcome variable and the animal grouping as the predictor variable. Metabolites were evaluated individually and separately for each tissue. All measured metabolite values were standardized (average = 0, standard deviation = 1). To account for multiple testing in the linear models, Bonferroni correction was firstly applied when metabolite with a *P*-value below the cutoff of *P* = 0.05 /’Number of metabolites after QC’ for each tissue were considered statistically different. This resulted in a cutoff of *P* < 1.42 × 10^–4^ for plasma (*P* < 0.05 / 351), *P* < 1.28 × 10^–4^ for liver (*P* < 0.05 / 391), *P* < 1.11 × 10^–4^ for kidney (*P* < 0.05 / 447). In addition to the Bonferroni correction, false discovery rate (FDR) levels (FDR < 0.05) and nominal significance levels (*P* < 0.05) were also considered as statistical significance as indicated.

For the human studies, potential risk factors and confounding parameters known to affect metabolite profiles were taken into account [13]. The basic model was adjusted for age and sex, while the full model included additional adjustments for body mass index (BMI), physical activity, high alcohol intake, smoking status, systolic blood pressure, HbA_1C_, fasting glucose levels, high-density lipoprotein cholesterol, triglycerides. Bonferroni correction was applied to account for multiple testing, and associations with a *P*-value below the cutoff of *P* < 0.05 /’Number of validated metabolites’ were considered statistically significant.

In the longitudinal study (S4 to F4), generalized estimating equations (GEE) were used to validate the significant associations in both the basic and fully adjusted models. We further employed a nearest-neighbor propensity score matching strategy with age and sex as covariates, testing various case–control ratios including 1:1, 1:2, 1:4, and 1:10, to achieve a balanced comparison between cases and controls.

All analyses were performed using R (version 4.0.3), a freely available software package developed by the R Core Team, 2022.

## Results

### Characteristics of the murine and human samples

In the mouse study, compared to WT mice, all three groups of diabetic mice (VG, MET, SGLT2i + MET) exhibited characteristic features of obesity, including higher body and liver weights (Table 1). However, the liver to body weight ratios were similar among all groups, suggesting proportional organ weight increases with body mass (Table 1).

**Table 1:** Characteristics of the murine samples

Clinical parameters	WT (n = 10)	VG (n = 10)	MET (n = 10)	SGLT2i + MET (n = 10)
Weight, g				
Body	22.0 (0.6)	47.9 (2.4)	47.8 (2.1)	46.8 (1.7)
Liver	1.02 (0.09)	2.56 (0.29)	2.61 (0.09)	2.36 (0.19)
Liver/Body, %	4.56 (0.42)	5.35 (0.51)	5.46 (0.28)	5.05 (0.43)
Kidney	0.16 (0.02)	0.20 (0.02)	0.21 (0.02)	0.21 (0.02)
Kidney/Body, %	0.73 (0.08)	0.43 (0.05)	0.45 (0.05)	0.46 (0.04)
Blood glucose, mg/dL				
6 weeks	108.8 (14.3)	442.5 (65.1)	454.8 (60.2)	439.1 (62.0)
8 weeks	106.7 (16.8)	421.6 (41.2)	322.6 (92.7)	129.9 (46.3)
Changed, %	1.9	4.7	29.1	70.1
Cholesterol, mg/dL				
HDL	84.3 (8.6)	125.3 (13.1)	135.5 (9.8)	156.0 (22.9)
LDL	14.5 (2.1)	18.76 (3.7)	19.49 (2.6)	25.19 (7.6)
Total	100.6 (12.2)	153.2 (16.1)	164.5 (12.6)	188.8 (29.9)
Triglycerides	122.2 (24.5)	224.8 (106.5)	262.4 (63.4)	199.9 (46.7)
Insulin, µg/l	1.03 (0.4)	7.76 (2.3)	7.86 (1.7)	6.78 (2.4)
C-reactive protein, mg/l	5.4 (1.1)	13.1 (3.3)	14.0 (4.1)	17.1 (3.7)

Means (standard deviation) of clinical variables in four mice groups

*WT* wild type mice, *VG* vehicle-gavaged diabetic mice, *MET* metformin-treated diabetic mice, *SGLT2i + MET* Sodium-glucose-cotransporter-2-inhibitor and metformin-treated diabetic mice, *HDL* high-density lipoprotein, *LDL* low-density lipoprotein

Diabetic mice also displayed hyperglycemia, as evidenced by elevated blood glucose and insulin levels. Dyslipidemia was observed with elevated cholesterol and triglyceride levels, and systemic inflammation was evident with elevated C-reactive protein levels (Table 1). Following two weeks of daily monotherapy with metformin or combination therapy in diabetic mice, significant reductions in blood glucose levels were observed. The MET mice showed a 29.1% reduction in blood glucose levels, while the SGLT2i + MET mice exhibited a substantial 70.0% reduction. In contrast, the VG and WT mice experienced modest reductions of 4.7% and 1.9%, respectively (Table 1). The SGLT2i + MET treatment led to lower body, insulin, and triglyceride levels compared to MET alone, but with higher cholesterol and C-reactive protein levels, suggesting a broader metabolic impact of combination therapy.

Cross-sectional validation of our findings from mouse studies was conducted in the KORA and QBB human studies, involving a total of 478 T2D patients and available metabolite profiles. The mt-T2D patients, compared to metformin-naïve T2D patients in both studies, were older and exhibited higher levels of HbA_1C_ and fasting glucose, suggesting more advanced dysglycemia in the mt-T2D group (Table 2).

**Table 2:** Characteristics of the KORA F4 and QBB cross-sectional study samples (N = 478)

Clinical parameters	KORA F4 study		QBB study	
	ndt-T2D (N = 114)	mt-T2D (N = 70)	ndt-T2D (N = 148)	mt-T2D (N = 146)
Age, years	65 (7.1)	66.1 (7.7)	46.3 (10.1)	52.5 (9.2)
Male, %	61	59	51,4	50,7
BMI, kg/m^2^	31.0 (4.8)	32.0 (5.6)	31.7 (6.3)	31.3 (6.1)
Physical active, % > 1 h per week	48	37	–	–
High alcohol intake^a^, %	25	19	–	–
Smoker, %	12	13	19.6	16.4
Systolic BP, mmHg	134.6 (19.1)	130.2 (18.1)	121.2 (16.6)	125.5 (15.5)
Diastolic BP, mmHg	77.9 (10.5)	74.8 (9.8)	78.2 (11.8)	76.5 (10.2)
HDL cholesterol, mg/dL	49.4 (11.5)	50.2 (9.7)	48.6 (13.6)	46.3 (11.0)
LDL cholesterol, mg/dL	136.6 (36.1)	123.2 (27.2)	121.5 (36.8)	102.8 (36.1)
Total cholesterol, mg/dL	214.4 (37.0)	201.8 (34.7)	201.1 (41.4)	181.2 (40.0)
Triglycerides, mg/dL	172.2 (129.3)	174.1 (141.0)	157.3 (88.4)	153.8 (65.6)
HbA_1C_, %	6.3 (0.9)	6.8 (1.1)	6.6 (1.3)	7.6 (1.6)
Fasting glucose, mg/dL	126.8 (30.5)	140.5 (34.9)	–	–
Metformin usage, %	0	100	0	100
Fasting (min. 8 h) before blood draw, %	100	100	27.7	27.4

Percentages of individuals or means (standard deviation) are shown for each variable and each group

*ndt-T2D* non-anti-diabetic drug-treated type 2 diabetes, *mt-T2D* metformin treated type 2 diabetes, *BMI* body mass index, *BP* blood pressure, *HDL* high-density lipoprotein, *LDL* low-density lipoprotein

^a^ ≥ 20 g/day for women; ≥ 40 g/day for men

Longitudinal validation was conducted in the prospective KORA study, spanning from the baseline survey (S4) to the 7-year follow-up (F4). Characteristics of these two groups, 34 T2D patients and 628 controls, were assessed at the S4 and F4 surveys (Table 3). The mt-T2D patients were found to be older, more sedentary, and had a higher prevalence of obesity compared to the group of 628 metformin-naïve individuals. Additionally, mt-T2D patients displayed higher blood pressure, triglyceride levels, and glycemic parameters at both surveys (Table 3).

**Table 3:** Characteristics of the KORA S4 → F4 prospective study samples (N = 662)

Clinical parameters	Baseline S4		Follow-up F4	
	w/o metformin	w/o metformin	w/o metformin	mt-T2D
N	628	34	628	34
Age, years	61.4 (4.3)	63.5 (3.8)	68.5 (4.3)	70.6 (3.9)
Male, %	51	50	51	50
BMI, kg/m^2^	27.9 (3.9)	32.4 (4.1)	28.2 (4.2)	31.8 (4.3)
Physical active^a^, %	48	32	57	44
High alcohol intake^b^, %	16	21	13	12
Smoker, %	13	9	8	3
Systolic BP, mmHg	132 (18.7)	145.5 (19.9)	128.2 (19.6)	131.5 (18.5)
Diastolic BP, mmHg	80 (10.1)	83.6 (9.8)	75.1 (9.9)	74.8 (9.9)
HDL cholesterol, mg/dL	59 (16.2)	53.9 (11.9)	56.7 (14.1)	52.3 (7.7)
LDL cholesterol, mg/dL	155.1 (40.9)	145.5 (38.4)	142.5 (36.5)	124.4 (23.6)
Total cholesterol, mg/dL	245.9 (42.0)	236.2 (41.5)	225.3 (40.4)	202.6 (33.8)
Triglyceride, mg/dL	130.3 (76.6)	170.2 (175.2)	132.4 (83.2)	148.3 (164.6)
HbA_1C_, %	5.6 (0.3)	6.3 (0.9)	5.6 (0.5)	6.5 (0.7)
Fasting glucose, mg/dL	99.7 (10.9)	127.6 (30.9)	100.8 (17.8)	126 (28.4)
Metformin usage, %	0	0	0	100
Fasting	100	100	100	100

Percentages or means (standard deviation) are shown for each variable and each group

*w/o* without, *BMI* body mass index, *BP* blood pressure, *HDL* high-density lipoprotein, *LDL* low-density lipoprotein

^a^ > 1 h per week

^b^ > 40 g/day in men; > 20 g/day in women

### Metformin effects on the blood metabolites in mouse and human studies

**Fig. 1:**
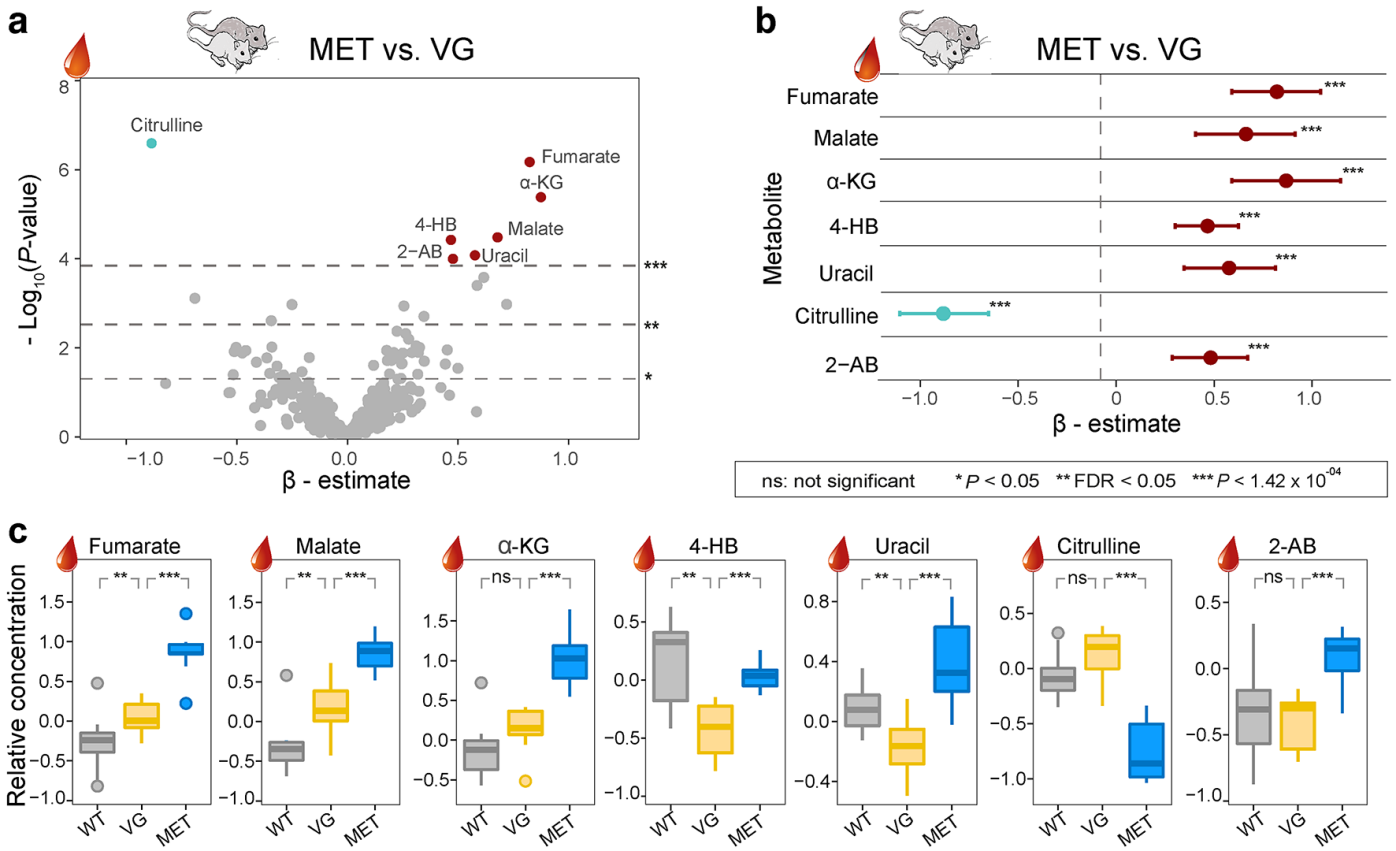
Effects of metformin and leptin receptor mutation in murine plasma. **a** Volcano plot of linear regression analysis result (β-estimate and *P* value) for 351 plasma metabolites in pairwise comparison of MET with VG diabetic mice. The upper, middle and lower dashed lines represent Bonferroni-corrected, FDR and nominal *(P* = 0.05) significance levels, respectively. **b** Seven metformin-associated metabolites of β-estimates with confidence intervals are shown. **c** Boxplots of seven metabolites in three groups. *WT* wild type mice, *VG* vehicle gavaged diabetic mice, *MET* metformin-treated diabetic mice, *FDR* false discovery rate, *2-AB* 2-aminobutyrate, *4-HB* 4-hydroxybutyrate, *α-KG* α-ketoglutarate. See also Supplementary Table 2, Additional File 1

In the mouse study, among the 351 metabolites analyzed, plasma levels of seven metabolites were significantly altered following two weeks of metformin treatment in db/db mice. These results were determined to be statistically significant after applying Bonferroni correction (Fig. 1a, Supplementary Table 2, Additional File 1). Out of the seven metformin-associated metabolites, three were identified as intermediates of the TCA cycle, namely fumarate, malate, and α-ketoglutarate (α-KG). Of these, six metabolites showed upregulation in response to metformin treatment (e.g., fumarate displayed a positive β-estimate in the linear regression analysis when comparing MET with VG mice as shown in Fig. 1b, and the relative concentration in MET mice was higher than that of VG mice as displayed with boxplots in Fig. 1c).

In the comparison of VG with WT mice, none of the seven metabolites exhibited a significant difference after applying Bonferroni correction. However, the values of four of these metabolites (fumarate, malate, 4-hydroxybutyrate [4-HB], and uracil) were nominally affected (*P* < 0.05) by the genetic background of the mice as well as HFD (Supplementary Table 2, Additional File 1). Interestingly, three metabolites (α-KG, citrulline, and 2-aminobutyrate [2-AB]) did not show a significant difference between VG and WT mice. This suggests that the changes induced by metformin in these three metabolites in db/db mice were independent of the physiological consequences of the leptin receptor mutation besides the HFD.

**Fig. 2:**
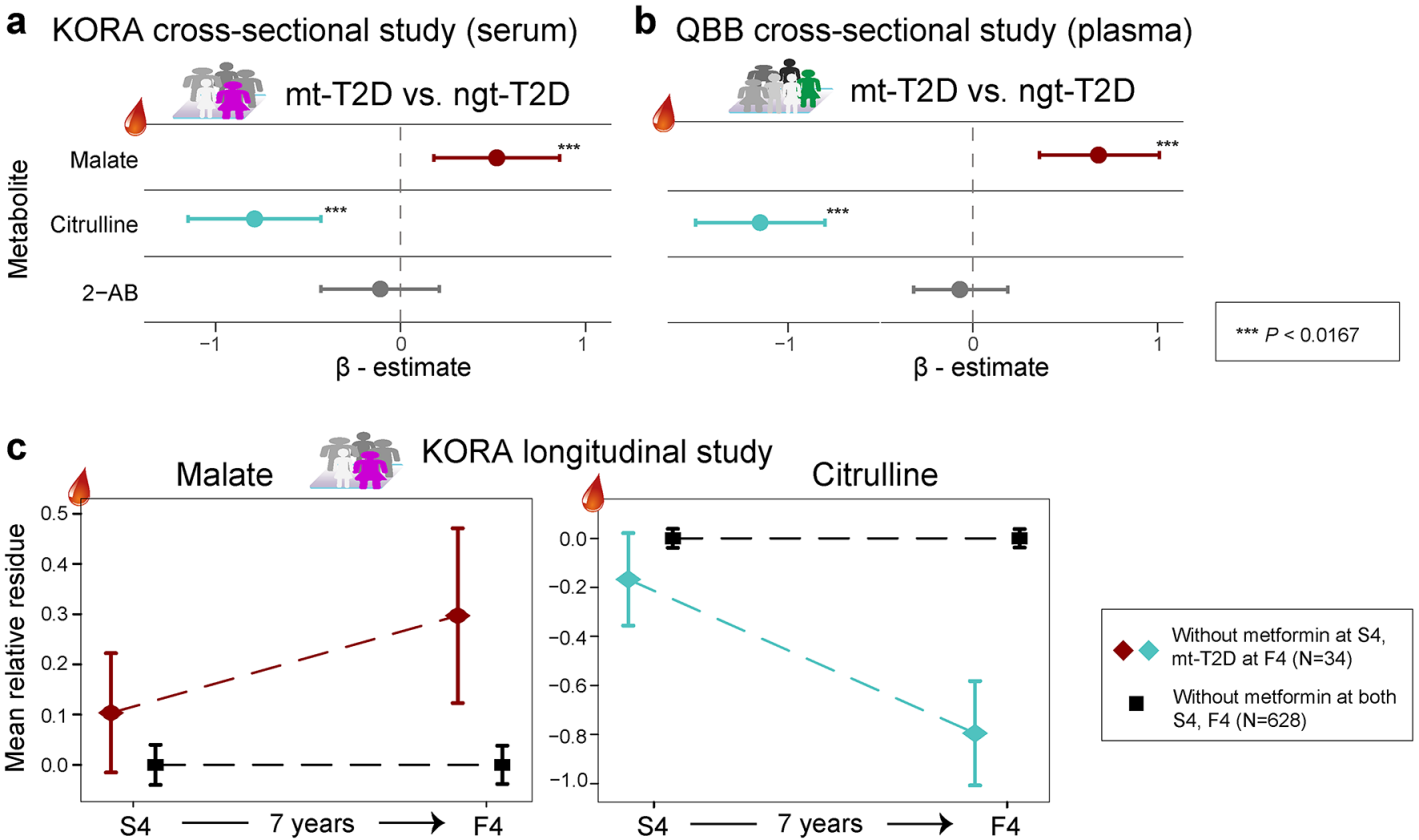
Cross-sectional and longitudinal analyses reveal specific pattern of metformin action in human serum and plasma. **a**, **b** β-estimates with confidence intervals of three metabolites in KORA and QBB cross-sectional human studies. **c** Mean relative residue of two metabolites in longitudinal KORA study. All analyses are based on the fully adjusted model (age, sex, BMI, physical activity, high alcohol intake, smoking status, systolic blood pressure, HbA_1C_, fasting glucose levels, high density lipoprotein cholesterol, triglycerides). *2-AB* 2 aminobutyrate. See also Supplementary Tables 3, 4, Additional File 1

In the human studies, three of the seven metformin-associated metabolites identified in murine plasma (malate, citrulline, and 2-AB) were measured in serum samples obtained from the KORA participants at both the S4 and F4 surveys. The analysis included a comparison between 70 individuals with mt-T2D and 114 individuals with ndt-T2D patients. Using a Bonferroni cutoff for significance (*P* < 0.017) for the three analyzed metabolites, two of them, citrulline and malate, were found to be significantly different in the fully adjusted model (Fig. 2a, Supplementary Table 3, Additional File 1). These findings were independently replicated in the plasma samples of patients from the QBB study, comparing 146 mt-T2D with 148 ndt-T2D patients, despite approximately 72% of the patients in the QBB study being non-fasting (Fig. 2b, Supplementary Table 3, Additional File 1). However, no significant correlation was observed for 2-AB in any of the comparisons (Fig. 2a, b, Supplementary Table 3, Additional File 1).

In the longitudinal KORA S4-F4 study, we specifically examined the impact of metformin on the serum levels of malate and citrulline (Fig. 2c). Our analysis confirmed that metformin use intiated after the baseline (S4) led to a significant increase in malate and a decrease in citrulline by the follow-up (F4). The changes were Bonferroni significant in basic model (malate: β = 0.39, *P* = 3.31 × 10^–4^), while nominal significant in the full model (malate: β = 0.25, *P* = 0.043), based on a comparison of 34 patients who started metformin treatment with 628 who did not (Supplementary Table 4a, Additional File 1).

Further validation through sensitivity analyses, which matched participants by age and sex across four case–control ratios, consistently showed Bonferroni significant results for both metabolites in all comparisons, except for malate in the ratio of 1:10 in the full model, which showed nominal significance, similar to the population-based study (Supplementary Tables 4b–e, Additional File 1). These sensitivity analyses further reinforce the robustness of the association between metformin use and alterations in malate and citrulline levels over time in different ratios of cases to controls.

### Metformin’s effects on the hepatic and renal metabolites

**Fig. 3:**
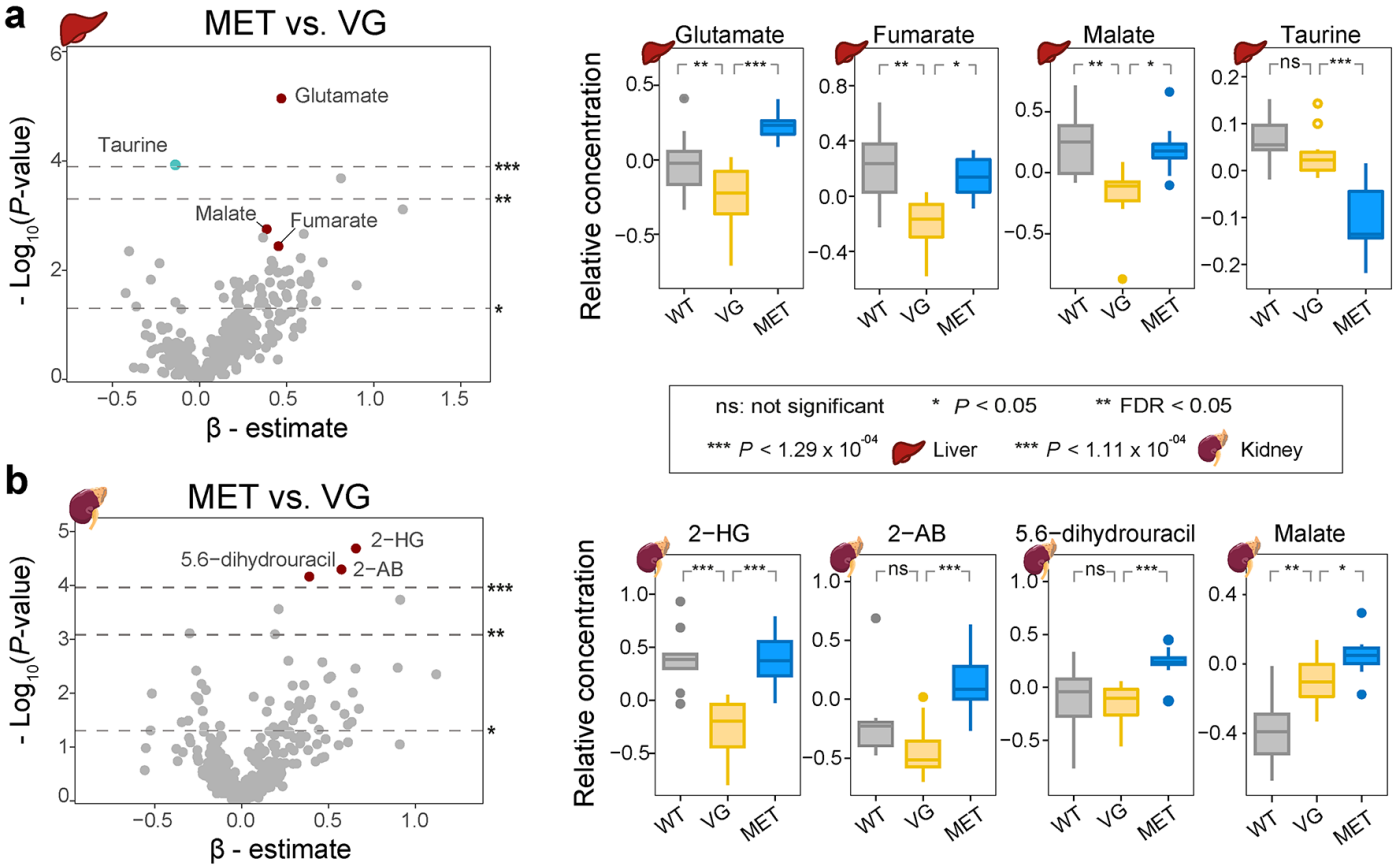
Hepatic and renal effects of metformin. Volcano plots of linear regression results for 391 hepatic (**a**) and 447 renal (**b**) metabolites for the comparison between MET and VG. The upper, middle and lower dashed lines represent Bonferroni-corrected, FDR and nominal (*P* = 0.05) significance levels, respectively. Boxplots of selected metformin-associated metabolites in WT, VG and MET mice are shown. *MET* metformin-treated db/db mice, *VG* vehicle-gavaged db/db mice, *WT* wild type mice, *2-AB* 2-aminobutyrate, *2-HG* 2-hydroxyglutarate. See also Supplementary Table 2, Additional File 1

Among the 391 analyzed metabolites in the liver, two metabolites, glutamate and taurine, showed Bonferroni-significant associations with metformin (Fig. 3a, Supplementary Table 2, Additional File 1). Metformin treatment resulted in an upregulation of glutamate and a downregulation of taurine compared to VG mice. Additionally, the values of fumarate and malate in the db/db liver were upregulated by metformin at nominal significance levels. In the comparison between VG and WT mice, glutamate, fumarate, and malate were downregulated at an FDR significant level, suggesting that these changes may be associated with the genetic background and the HFD. Notably, the observed up and down regulation of these metabolites (glutamate, fumarate, and malate) among the MET, VG and WT mice may indicate beneficial effects of metformin in the liver of the db/db mice.

Moving to the kidneys of db/db mice, among the 447 analyzed metabolites, three metabolites (2-hydroxyglutarate [2-HG], 2-AB, and 5,6-dihydrouracil) exhibited Bonferroni-significant upregulation due to metformin treatment (Fig. 3b, Supplementary Table 2, Additional File 1). Additionally, malate values in the db/db kidneys were altered by metformin at a nominal significant level. In the comparison between VG and WT mice, 2-HG had a Bonferroni-significant downregulation, malate showed an upregulation at a FDR significant level, while comparable levels of 2-AB and 5,6-dihydrouracil were observed between VG and WT mice.

### Metabolic effects of adding SGLT2i to metformin

**Fig. 4:**
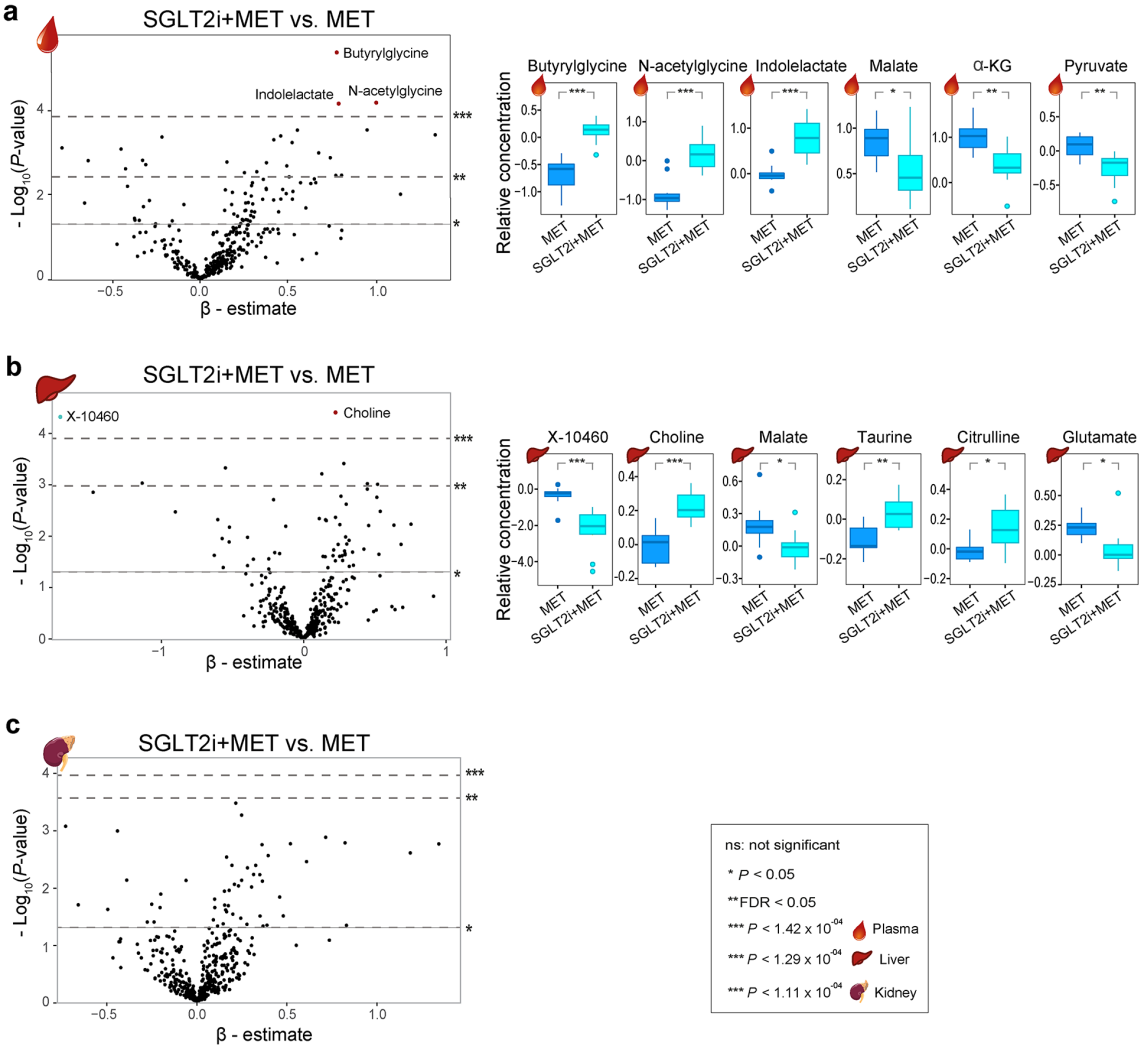
Metabolic effects of combination therapy in the three murine tissues. Volcano plots in plasma (**a**), liver (**b**) and kidney (**c**) when compare SGLT2i + MET with MET mice. The upper, middle and the lower dashed lines represent Bonferroni-corrected, FDR and nominal (*P* = 0.05) significance levels, respectively. Boxplots of selected metabolites in MET and SGLT2i + MET mice are shown. *MET* metformin-treated db/db mice, *SGLT2i + MET* SGLT2i and metformin treated db/db mice. See also Supplementary Table 5, Additional File 1

Of 716 analyzed metabolites in the three tissues, three (butyrylglycine, N-acetyl glycine, and indole lactate) in plasma and two (choline and X-10460) in the liver were found to have Bonferroni-significant associations with the combination therapy when comparing SGLT2i + MET with MET mice (Fig. 4a, b, Supplementary Table 5, Additional File 1). Except for X-10460, all four identified metabolites were upregulated in the combination therapy group. However, none of the 447 analyzed renal metabolites showed either Bonferroni or FDR significant differences in the pairwise comparison between SGLT2i + MET and MET mice (Fig. 4c).

In addition to the Bonferroni-significant metabolites associated with the combination therapy, several metabolites related to the TCA cycle showed FDR or nominal significant alterations. In the pairwise comparison between SGLT2i + MET and MET mice, the levels of malate, α-KG, and pyruvate in plasma, malate and glutamate in the liver, were downregulated. On the other hand, taurine and citrulline in the liver were upregulated (Fig. 4a, b, Supplementary Table 5, Additional File 1).

### Tissue- and drug-specific effects of TCA cycle metabolites and its anaplerosis

**Fig. 5:**
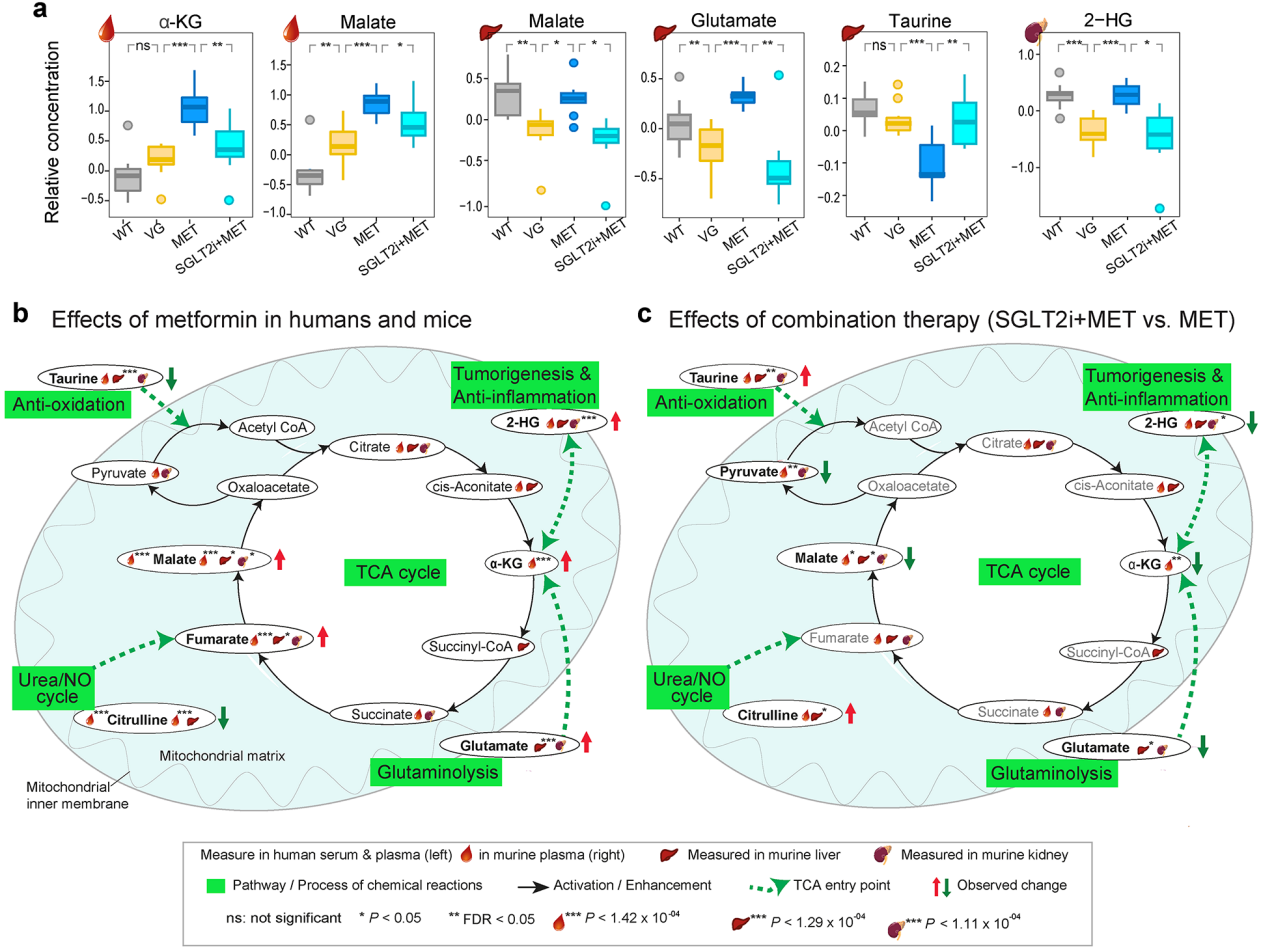
Alteraration of TCA cycle metabolites and related pathways. **a** Boxplots showing selected metabolites across four db/db mouse groups. **b** Schematic overview of metformin’s effects on TCA cycle related metabolites in liver, kidney, and plasma of db/db mice and serum/plasma in humans. **c** Schematic of combined SGLT2i and metformin therapy in the same mouse tissues. This figure provides a simplified overview intended to facilitate general understanding of the alterations in TCA cycle metabolites across different tissues and treatment modalities. It is not meant to depict precise metabolic flows or fully detailed pathway interactions. Each organ has unique metabolic functions; thus, interpretations should consider the specific metabolic context of each tissue. *2-HG* 2-hydroxyglutarate, *α-KG* α-ketoglutarate, *TCA* citric acid, *NO* nitric oxide

Collectively, we observed different tissue-dependent responses to metformin and its combination with SGLT2i for intermediates of TCA cycle and its anaplerosis. Specifically, circulating malate levels in all three db/db mice (VG, MET, and SGLT2i + MET) were higher than WT ones, whereas their hepatic values were comparable between WT and MET mice, but lowered in both VG and SGLT2i + MET mice (Fig. 5a). These observations may indicate that the TCA cycle activity in leukocytes (erythrocytes lack mitochondria and functional TCA cycle) and hepatocytes responds differently to metformin. Moreover, in the three group of db/db mice, similar patterns for circulating malate and α-KG, hepatic malate and glutamate, and renal 2-HG, were observed (e.g., highest levels were observed in MET mice). Whereas, taurine levels were lowest in the MET mice in the liver (Fig. 5a). These results suggested that add-on SGLT2i to metformin reversed abundances of these metabolites, thereby suggesting a bidirectional modulation of TCA cycle metabolites and anaplerosis.

## Discussion

Our comprehensive study has explored the impact of metformin and SGLT2i on 716 distinct metabolites across the liver, kidneys, and blood, using both animal models and human study participants. Our key findings highlight significant alterations in the TCA cycle metabolites caused by metformin, whose effects are modulated when combined with SGLT2i, resulting in a bidirectional'reversal' of its core impacts on energy metabolism (Fig. 5b and c).

The clinical relevance of our study was established by confirming similar metabolic alterations, specifically, increased malate and decreased citrulline, in T2D patients from diverse backgrounds. These consistent findings emphasize metformin's role in modulating key metabolic processes and have implications for the treatment of T2D, indicating potential areas for therapeutic refinement.

The TCA cycle relies on intermediate metabolites like α-KG, fumarate, and malate, which are consumed during energy production and replenished by anaplerotic pathways [28]. Our analysis encompasses four of these pathways, including the glutaminolysis process that transforms glutamine into glutamate and ultimately into α-KG. Our observed upregulated hepatic glutamate by metformin could lead to elevated circulating α-KG levels, and the drug's association with upregulated renal 2-HG levels. Additionally, metformin's influence on fumarate and malate levels may link to changes in the urea and NO cycles. All of our results point to broader impacts of metformin in cancer cells, affecting insulin secretion and immune cell modulation [29–31]. Investigating metformin's influence on leukocyte and hepatocyte TCA cycle dynamics is an area for future study.

α-KG plays a critical role beyond the TCA cycle, impacting epigenetic regulation and immune response [28]. Notably, while metformin did not affect succinate levels, a key cellular signaling metabolite, it did increase 2-HG levels in the kidneys of diabetic mice, suggesting a protective role against diabetic kidney disease (DKD).

2-HG, a structural analog of α-KG, also known as an "oncometabolite", plays a complex role in regulating immune responses and metabolic pathways, including mTOR [32–36]. Its modulation by metformin could offer renal benefits through its anti-inflammatory and antifibrotic properties, despite the different actions of its isomers, L-2-HG and D-2-HG [28].

The observed downregulation in hepatic taurine levels post-metformin treatment prompts further exploration of its effects on antioxidant defenses [37]. Research in rat models has demonstrated that taurine can modulate the activity of the pyruvate dehydrogenase complex, thereby influencing the rate of pyruvate's entry, or anaplerosis, into the TCA cycle [38]. Despite taurine's known role in bolstering the antioxidative actions of metformin [39], taurine supplementation has been recommended for conditions such as congestive heart failure [40]. This discrepancy suggests a complex interplay between metformin's therapeutic effects and its influence on metabolic pathways, warranting further investigation to elucidate the specific mechanisms at play.

Conversely, combining metformin with SGLT2i presented a contrasting metabolic profile. This regimen not only altered the levels of plasma α-KG, hepatic glutamate, renal 2-HG, and both plasma and hepatic malate, but also restored hepatic taurine levels decreased by metformin alone. These changes indicate that SGLT2i may counterbalance metformin's metabolic actions, potentially affecting anaplerotic flux and physiological processes. The reduction in renal 2-HG levels could signify a subdued immune response, as indicated by elevated C-reactive protein levels in all three groups of diabetic mice as presented in Table 1.

Mechanistically, SGLT2i have been shown to reduce glutamate dehydrogenase (GDH) activity, affecting NADH production and thus the mitochondrial electron transport chain (ETC), essential for ATP generation via oxidative phosphorylation (OXPHOS) [41]. This suppression may also elevate the AMP/ATP ratio, activating AMP-activated protein kinase (AMPK), a key target of metformin that promotes fatty acid breakdown and has beneficial effects on liver diseases [42], including metabolic dysfunction-associated steatotic liver disease (MASLD) [43]. Excessively elevated hepatic mitochondrial TCA cycle activity has been observed in HFD-induced fatty liver and in patients with MASLD [44, 45]. In these cases, oxidative substrates are produced, leading to oxidative stress and tissue damage in the liver. SGLT2i's inhibition of GDH may protect against MASLD by attenuating an overactive TCA cycle, thereby reducing oxidative stress, as suggested by observations in diabetic mice kidneys and liver [46].

In summary, our findings reveal that metformin and SGLT2i therapy modulate several metabolic pathways, with implications for TCA cycle dynamics, immune response, and antioxidant defenses in diabetes management. These insights provide a foundation for further exploration of the potential drug-drug interactions and the metabolic effects of these therapies on liver diseases and diabetic complications.

## Limitations

Our non-targeted metabolomics analysis utilized 716 metabolites but faced limitations due to the inclusion of unknown metabolites like X-10460, whose role in SGLT2i + MET therapy remains to be elucidated. We selected AVE2268 as the representative SGLT2i due to its availability, despite its limited clinical use. Nonetheless, its cardiovascular benefits appear to be consistent with those of other SGLT2 inhibitors, suggesting a potential class effect [47]. We chose to use mice in the 6-week age group for this study because they develop diabetes earlier than mature mice, allowing us to investigate early changes associated with diabetes. However, a limitation is that the tissues from mice at the time of sacrifice (8 weeks old) are not fully mature, potentially affecting the results. Additionally, tissue hypoxia induced during sacrifice might have influenced the overall metabolic effects observed in our mouse study. Although our findings in mice were corroborated with human blood samples, data from patients undergoing SGLT2i therapy were unavailable due to the timing of our study relative to SGLT2i approval. The study's exclusive focus on male mice may also limit the generalizability of the results. Moreover, our steady-state metabolomics approach does not capture the dynamics of metabolic pathways, including the TCA cycle and associated anaplerotic processes. Future studies should consider employing targeted analyses to gain a deeper understanding of these complex metabolic interactions.

## Conclusion

Our research underscores the complex effects of metformin and SGLT2i on metabolic processes, extending beyond traditional diabetes management. Metformin may reduce inflammation, while SGLT2 inhibitors could help normalize TCA cycle activity, benefiting conditions like MASLD and affecting immune cell function.

These findings highlight the need for further research to explore how these drugs modify metabolism and to develop targeted treatments. Additionally, our insights suggest potential for personalized treatment strategies that tailor therapeutic outcomes to individual metabolic profiles.

Overall, our study points to the broader potential of these medications in treating complex metabolic and immune-mediated disorders, reflecting their evolving role in modern medicine.

### Supplementary Information

Additional File 1 - Supplementary Table 1. Utilized 716 metabolites in three murine tissues. Supplementary Table 2. Metformin-associated metabolites in murine plasma, liver and kidney. Supplementary Table 3. Corroboration of metformin-associated metabolites in human studies. Supplementary Table 4. Validation of two metabolites in longitudinal KORA S4/F4 study. Supplementary Table 5. Combination therapy altered metabolites in murine plasma, liver and kidney. Additional File 2 - Detailed description of the untargeted method. Supplementary Material 1

## Data Availability

The KORA S4/F4 data sets are not publicly available because of data protection agreements but can be provided upon request through the KORA-PASST (Project application self-service tool, www.helmholtz-muenchen.de/kora-gen). The QBB data can be obtained to the researcher to submit access application forms online (https://researchportal.qatarbiobank.org.qa/).

## Abbreviations

2-AB: 2-Aminobutyrate
2-HG: 2-Hydroxyglutarate
4-HB: 4-Hydroxybutyrate
α-KG: α-Ketoglutarate
AMP: Adenosine monophosphate
AMPK: AMP-activated protein kinase
ATP: Adenosine triphosphate
BMI: Body mass index
BP: Blood pressure
DKD: Diabetic kidney disease
eNOS: Endothelial nitric oxide synthase
ETC: Electron transport chain
FDR: False discovery rate
GDH: Glutamate dehydrogenase
HDL: High-density lipoprotein
HFD: High-fat diet
LDL: Low-density lipoprotein
MET: Metformin-treated diabetic mice
mTOR: Mammalian target of rapamycin
mt-T2D: Metformin treated type 2 diabetes
NAD: Nicotinamide adenine dinucleotide
NADH: Nicotinamide adenine dinucleotide reduced form
MASLD: Metabolic dysfunction-associated steatotic liver disease
ndt-T2D: Non-anti-diabetic drug-treated type 2 diabetes
NO: Nitric oxide
OXPHOS: Oxidative phosphorylation
QC: Quality control
SGLT2i: Sodium-glucose-cotransporter-2 inhibitors
SGLT2i + MET: Sodium-glucose-cotransporter-2-inhibitor and metformin-treated diabetic mice
T2D: Type 2 diabetes
TCA: Citric acid, also known as tricarboxylic acid
w/o: Without
WT: Wild type mice
VG: Vehicle gavaged diabetic mice
