# Supplementary material for: Bidirectional modulation of TCA cycle metabolites and anaplerosis by metformin and its combination with SGLT2i
Source: Cardiovasc Diabetol. 2024 Jun 12;23:199. doi: 10.1186/s12933-024-02288-x (PMC11170891; doi:10.1186/s12933-024-02288-x)
Supplement: Supplementary file 1 — Additional File 1 - Supplementary Table 1. Utilized 716 metabolites in three murine tissues. Supplementary Table 2. Metformin-associated metabolites in murine plasma, liver and kidney. Supplementary Table 3. Corroboration of metformin-associated metabolites in human studies. Supplementary Table 4. Validation of two metabolites in longitudinal KORA S4/F4 study. Supplementary Table 5. Combination therapy altered metabolites in murine plasma, liver and kidney. Additional File 2 - Detailed description of the untargeted method. Supplementary Material 1 [file 12933_2024_2288_MOESM1_ESM.docx]

**Bidirectional modulation of TCA cycle metabolites and anaplerosis by metformin and its combination with SGLT2i**

Additional File 1

**Supplementary Table 1.** Utilized 716 metabolites in three murine tissues

**Supplementary Table 2.** Metformin-associated metabolites in murine plasma, liver and kidney

**Supplementary Table 3.** Corroboration of metformin-associated metabolites in human studies

**Supplementary Table 4.** Validation of two metabolites in longitudinal KORA S4/F4 study

**Supplementary Table 5.** Combination therapy altered metabolites in murine plasma, liver and kidney

Additional File 2

**Detailed description of the untargeted method**

**Supplementary Table 1.** **Utilized 716 metabolites in three murine tissues**

The Metabolon IDs and biochemical names of 716 metabolites that were used in at least one tissue, are shown in the first and second column. Metabolite names beginning with an ‘X-’ are denoting structurally unknown detected metabolites. Column three and five show the status for this project (used = ’x’)/excluded = ’-’). Stars (*) denote metabolites that were not confirmed with a standard at the time of discovery. Abbreviations: P: plasma, L: liver, K: kidney.

| **Metabolite ID** | **Metabolite name** | **Plasma** | **Liver** | **Kidney** |
| --- | --- | --- | --- | --- |
| n = 136 |  |  |  |  |
| M00053 | glutamine | x | x | x |
| M00054 | tryptophan | x | x | x |
| M00059 | histidine | x | x | x |
| M00060 | leucine | x | x | x |
| M00063 | cholesterol | x | x | x |
| M00064 | phenylalanine | x | x | x |
| M00485 | spermidine | x | x | x |
| M00512 (L) / M11398 (P,K) | asparagine | x | x | x |
| M00514 | cytidine | x | x | x |
| M00527 | lactate | x | x | x |
| M00542 | 3-hydroxybutyrate (BHBA) | x | x | x |
| M31266 | fructose | x | x | x |
| M00584 | mannose | x | x | x |
| M00594 | nicotinamide | x | x | x |
| M00605 | uracil | x | x | x |
| M00606 | uridine | x | x | x |
| M01105 | linoleate (18:2n6) | x | x | x |
| M01107 | allantoin | x | x | x |
| M01110 | arachidonate (20:4n6) | x | x | x |
| M01121 | margarate (17:0) | x | x | x |
| M01125 | isoleucine | x | x | x |
| M01126 | alanine | x | x | x |
| M01284 | threonine | x | x | x |
| M01299 | tyrosine | x | x | x |
| M01301 | lysine | x | x | x |
| M01302 | methionine | x | x | x |
| M01303 | malate | x | x | x |
| M01336 | palmitate (16:0) | x | x | x |
| M01358 | stearate (18:0) | x | x | x |
| M01359 | oleate (18:1n9) | x | x | x |
| M01365 | myristate (14:0) | x | x | x |
| M01444 | pipecolate | x | x | x |
| M01493 | ornithine | x | x | x |
| M01494 | 5-oxoproline | x | x | x |
| M01497 (L,K) / M34285 (P) | ethanolamine | x | x | x |
| M01508 | pantothenate | x | x | x |
| M01561 | alpha-tocopherol | x | x | x |
| M01564 | citrate | x | x | x |
| M01572 | glycerate | x | x | x |
| M01589 | N-acetylmethionine | x | x | x |
| M01604 | urate | x | x | x |
| M01642 | caprate (10:0)\|caprate100 | x | x | x |
| M01643 | fumarate | x | x | x |
| M01645 | laurate (12:0) | x | x | x |
| M01648 | serine | x | x | x |
| M01649 | valine | x | x | x |
| M01670 | urea | x | x | x |
| M02125 (K) / M32975 (P,L) | taurine | x | x | x |
| M02134 | flavin adenine dinucleotide (FAD) | x | x | x |
| M03141 | betaine | x | x | x |
| M04966 | xylitol | x | x | x |
| M06146 | 2-aminoadipate | x | x | x |
| M11777 | glycine | x | x | x |
| M12035 | pelargonate (9:0) | x | x | x |
| M15053 | sorbitol | x | x | x |
| M15122 | glycerol | x | x | x |
| M15365 | glycerol 3-phosphate (G3P) | x | x | x |
| M15500 | carnitine | x | x | x |
| M15990 | glycerophosphorylcholine (GPC) | x | x | x |
| M17805 | dihomo-linoleate (20:2n6) | x | x | x |
| M18362 | azelate (nonanedioate) | x | x | x |
| M18369 | gamma-glutamylleucine | x | x | x |
| M18467 | eicosapentaenoate (EPA; 20:5n3) | x | x | x |
| M18497 | taurocholate | x | x | x |
| M19323 | docosahexaenoate (DHA; 22:6n3) | x | x | x |
| M19324 | 1-stearoylglycerophosphoinositol | x | x | x |
| M19934 | myo-inositol | x | x | x |
| M20488 (L,K) / M31263 (P) | glucose | x | x | x |
| M20699 | erythritol | x | x | x |
| M21044 | 2-hydroxybutyrate (AHB) | x | x | x |
| M21127 | 1-palmitoylglycerol (1-monopalmitin) | x | x | x |
| M21184 | 1-oleoylglycerol (1-monoolein) | x | x | x |
| M27718 | creatine | x | x | x |
| M27728 | glycerol 2-phosphate | x | x | x |
| M31453 | cysteine | x | x | x |
| M31555 | pyridoxate | x | x | x |
| M31850 | butyrylglycine | x | x | x |
| M32198 | acetylcarnitine | x | x | x |
| M32328 (P) / M32489 (L,K) | hexanoylcarnitine\|caproate (6:0)\|caproate60 | x | x | x |
| M32342 | adenosine 5'-monophosphate (AMP) | x | x | x |
| M32393 | gamma-glutamylvaline | x | x | x |
| M32418 | myristoleate (14:1n5) | x | x | x |
| M32452 | propionylcarnitine | x | x | x |
| M32504 | docosapentaenoate (n3 DPA; 22:5n3) | x | x | x |
| M32635 | 1-linoleoylglycerophosphoethanolamine* | x | x | x |
| M32654 | X - 21365 (N,N,N-trimethyl-5-aminovalerate) | x | x | x |
| M32815 | 2-arachidonoylglycerophosphoethanolamine* | x | x | x |
| M32980 | adrenate (22:4n6) | x | x | x |
| M33447 | palmitoleate (16:1n7) | x | x | x |
| M33454 | gulono-1,4-lactone | x | x | x |
| M33477 | erythronate* | x | x | x |
| M33510 | X - 12095\|X - 12095\|N1-methyl-3-pyridone-4-carboxamide | x | x | x |
| M33587 | eicosenoate (20:1n9 or 11) | x | x | x |
| M33955 | 1-palmitoylglycerophosphocholine | x | x | x |
| M33960 | 1-oleoylglycerophosphocholine | x | x | x |
| M33961 | 1-stearoylglycerophosphocholine | x | x | x |
| M33969 | stearidonate (18:4n3) | x | x | x |
| M33971 | 10-heptadecenoate (17:1n7) | x | x | x |
| M33972 | 10-nonadecenoate (19:1n9) | x | x | x |
| M33983 | tauro-beta-muricholate | x | x | x |
| M34035 | linolenate [alpha or gamma; (18:3n3 or 6)] | x | x | x |
| M34416 | 1-stearoylglycerophosphoethanolamine | x | x | x |
| M34419 | 1-linoleoylglycerophosphocholine | x | x | x |
| M34592 | ophthalmate | x | x | x |
| M35186 | 1-arachidonoylglycerophosphoethanolamine* | x | x | x |
| M35256 | 2-arachidonoylglycerophosphocholine* | x | x | x |
| M35257 | 2-linoleoylglycerophosphocholine* | x | x | x |
| M35628 | 1-oleoylglycerophosphoethanolamine | x | x | x |
| M35631 | 1-palmitoylglycerophosphoethanolamine | x | x | x |
| M35718 | dihomo-linolenate (20:3n3 or n6) | x | x | x |
| M36593 | 2-linoleoylglycerophosphoethanolamine* | x | x | x |
| M36768 | valerylglycine | x | x | x |
| M37253 | X - 05491\|2-hydroxyglutarate | x | x | x |
| M37478 | docosapentaenoate (n6 DPA; 22:5n6) | x | x | x |
| M37506 | X - 10419\|palmitoyl sphingomyelin | x | x | x |
| M37516 | arabonate | x | x | x |
| M37752 | 13-HODE + 9-HODE | x | x | x |
| M32644 | X - 11327 | x | x | x |
| M32729 | X - 11412 | x | x | x |
| M12774 | X - 03094 | x | x | x |
| M22032 | X - 08766 | x | x | x |
| M22494 | X - 08994 | x | x | x |
| M27890 | X - 10611 | x | x | x |
| M32850 | X - 11533 | x | x | x |
| M32859 | X - 11542 | x | x | x |
| M32894 | X - 10445 | x | x | x |
| M32957 | X - 11640 | x | x | x |
| M33220 | X - 11875 | x | x | x |
| M33627 | X - 12206 | x | x | x |
| M33892 | X - 12450 | x | x | x |
| M33910 | X - 12465 | x | x | x |
| M35754 | X - 13859 | x | x | x |
| M35767 | X - 13872 | x | x | x |
| M37364 | X - 15222 | x | x | x |
| M38966 | X - 16397 | x | x | x |
| M39154 | X - 16581 | x | x | x |
| n = 27 |  |  |  |  |
| M00396 | glutarate (pentanedioate) | x | x | - |
| M01356 | nonadecanoate (19:0) | x | x | - |
| M02132 | citrulline | x | x | - |
| M12025 | cis-aconitate | x | x | - |
| M15506 | choline | x | x | - |
| M15686 | beta-hydroxypyruvate | x | x | - |
| M15720 | N-acetylglutamate | x | x | - |
| M17945 | 2-hydroxystearate | x | x | - |
| M27727 (L) / M38783 (P) | glutathione, oxidized (GSSG) | x | x | - |
| M32197 | 3-(4-hydroxyphenyl)lactate | x | x | - |
| M32350 | 1-methylimidazoleacetate | x | x | - |
| M32415 | docosadienoate (22:2n6) | x | x | - |
| M33228 | 1-arachidonoylglycerophosphocholine* | x | x | - |
| M33822 | 1-docosahexaenoylglycerophosphocholine* | x | x | - |
| M34214 | 1-arachidonoylglycerophosphoinositol* | x | x | - |
| M35159 | cysteine-glutathione disulfide | x | x | - |
| M35688 | 2-palmitoylglycerophosphoethanolamine* | x | x | - |
| M36594 | 1-linoleoylglycerophosphoinositol* | x | x | - |
| M38165 | palmitoyl ethanolamide | x | x | - |
| M19414 | X - 06350 | x | x | - |
| M32855 | X - 11538 | x | x | - |
| M33231 | X - 11886 | x | x | - |
| M33403 | X - 12051 | x | x | - |
| M35072 | X - 13372 | x | x | - |
| M38775 | X - 16209 | x | x | - |
| M39151 | X - 16578 | x | x | - |
| M39153 | X - 16580 | x | x | - |
| n = 109 |  |  |  |  |
| M00055 | beta-alanine | - | x | x |
| M00057 | glutamate | - | x | x |
| M00554 | adenine | - | x | x |
| M00555 | adenosine | - | x | x |
| M00587 | gluconate | - | x | x |
| M00590 | hypotaurine | - | x | x |
| M00597 | phosphoenolpyruvate (PEP) | - | x | x |
| M01123 | inosine | - | x | x |
| M01408 | putrescine | - | x | x |
| M01414 | 3-phosphoglycerate | - | x | x |
| M01416 | gamma-aminobutyrate (GABA) | - | x | x |
| M01419 | 5-methylthioadenosine (MTA) | - | x | x |
| M01481 | inositol 1-phosphate (I1P) | - | x | x |
| M01573 | guanosine | - | x | x |
| M01585 | N-acetylalanine | - | x | x |
| M12102 | phosphoethanolamine | - | x | x |
| M01640 | ascorbate (Vitamin C) | - | x | x |
| M01651 | pyridoxal | - | x | x |
| M01827 | riboflavin (Vitamin B2) | - | x | x |
| M01898 | proline | - | x | x |
| M02078 | pyrophosphate (PPi) | - | x | x |
| M03127 | hypoxanthine | - | x | x |
| M03147 | xanthine | - | x | x |
| M05278 | nicotinamide adenine dinucleotide (NAD+) | - | x | x |
| M35727 (L,K) | 1,2-dipalmitoylglycerol | - | x | x |
| M12080 (L) / M12083 (K) | ribose | - | x | x |
| M15136 | xanthosine | - | x | x |
| M15443 | glucuronate | - | x | x |
| M15772 (L,K) / M38075 (K) | arabitol\|ribitol | - | x | x |
| M15948 | S-adenosylhomocysteine (SAH) | - | x | x |
| M15996 | aspartate | - | x | x |
| M18374 | methionine sulfoxide | - | x | x |
| M18534 | glucosamine | - | x | x |
| M19503 | stearoyl sphingomyelin | - | x | x |
| M22171 | glycylproline | - | x | x |
| M32553 | phenol sulfate | - | x | x |
| M32675 | C-glycosyltryptophan* | - | x | x |
| M34396 | choline phosphate | - | x | x |
| M34398 | glycylleucine | - | x | x |
| M35092 | 7-beta-hydroxycholesterol | - | x | x |
| M35305 | 1-palmitoylglycerophosphoinositol* | - | x | x |
| M35433 | hydroxyisovaleroyl carnitine | - | x | x |
| M35439 | glutaroyl carnitine | - | x | x |
| M35638 | xylonate | - | x | x |
| M35675 | 2-hydroxypalmitate | - | x | x |
| M35683 (K) / M35687 (L) | 2-oleoylglycerophosphoethanolamine* | - | x | x |
| M35692 | 7-alpha-hydroxycholesterol | - | x | x |
| M35728 | 1,3-dipalmitoylglycerol | - | x | x |
| M35963 | 1,3-dihydroxyacetone | - | x | x |
| M36738 | gamma-glutamylglutamate | - | x | x |
| M37060 | methylglutaroylcarnitine | - | x | x |
| M37123 | N-glycolylneuraminate | - | x | x |
| M37459 | X - 11568\|ergothioneine | - | x | x |
| M37538 | 15-HETE | - | x | x |
| M40008 | isoleucylglycine | - | x | x |
| M16028 | X - 03998 | - | x | x |
| M16116 | X - 04051 | - | x | x |
| M17989 | X - 05229 | - | x | x |
| M19110 | X - 05978 | - | x | x |
| M19602 | X - 06666 | - | x | x |
| M19603 | X - 06667 | - | x | x |
| M21391 | X - 08167 | - | x | x |
| M21394 | X - 08169 | - | x | x |
| M27256 | X - 10500 | - | x | x |
| M31360 | X - 10875 | - | x | x |
| M31361 | X - 10876 | - | x | x |
| M32518 | X - 11204 | - | x | x |
| M32892 | X - 11575 | - | x | x |
| M32895 | X - 11578 | - | x | x |
| M32902 | X - 11585 | - | x | x |
| M32913 | X - 11596 | - | x | x |
| M32930 | X - 11613 | - | x | x |
| M32932 | X - 11615 | - | x | x |
| M32974 | X - 11612 | - | x | x |
| M33014 | X - 10457 | - | x | x |
| M33015 | X - 11677 | - | x | x |
| M33031 | X - 11687 | - | x | x |
| M33163 | X - 11818 | - | x | x |
| M33884 | X - 12442\|5,8-tetradecadienoate | - | x | x |
| M34221 | X - 12627 | - | x | x |
| M34265 | X - 12660 | - | x | x |
| M34268 | X - 12663 | - | x | x |
| M34841 | X - 13149 | - | x | x |
| M35270 | X - 13496 | - | x | x |
| M35335 | X - 13557 | - | x | x |
| M35464 | X - 13671 | - | x | x |
| M35646 | X - 13819 | - | x | x |
| M36931 | X - 14904 | - | x | x |
| M37277 | X - 15136 | - | x | x |
| M37397 | X - 15255 | - | x | x |
| M37559 | X - 15312 | - | x | x |
| M37663 | X - 15415 | - | x | x |
| M37755 | X - 15507 | - | x | x |
| M37763 | X - 15515 | - | x | x |
| M37770 | X - 15522 | - | x | x |
| M37776 | X - 15528 | - | x | x |
| M37801 | X - 15552 | - | x | x |
| M38363 | X - 15933 | - | x | x |
| M38505 | X - 16063 | - | x | x |
| M38546 | X - 16103 | - | x | x |
| M38817 | X - 16249 | - | x | x |
| M39180 | X - 16607 | - | x | x |
| M39186 | X - 16613 | - | x | x |
| M39187 | X - 16614 | - | x | x |
| M39189 | X - 16616 | - | x | x |
| M39190 | X - 16617 | - | x | x |
| M39641 | X - 17010 | - | x | x |
| M39648 | X - 17017 | - | x | x |
| M39650 | X - 17019 | - | x | x |
| n=70 |  |  |  |  |
| M00513 | creatinine | x | - | x |
| M00575 (P) / M15835 (K) | arabinose\|xylose | x | - | x |
| M00599 | pyruvate | x | - | x |
| M01366 | trans-4-hydroxyproline | x | - | x |
| M01437 | succinate | x | - | x |
| M01577 (K) / M32348 (P) | 2-aminobutyrate | x | - | x |
| M01598 | tigloylglycine | x | - | x |
| M01638 | arginine | x | - | x |
| M03155 | 3-ureidopropionate | x | - | x |
| M05086 | alpha-ketobutyrate | x | - | x |
| M11438 | phosphate | x | - | x |
| M12067 | undecanoate (11:0) | x | - | x |
| M15140 | kynurenine | x | - | x |
| M15650 | N1-methyladenosine | x | - | x |
| M15676 | 3-methyl-2-oxovalerate | x | - | x |
| M15737 | glycolate (hydroxyacetate) | x | - | x |
| M15821 | fucose | x | - | x |
| M15949 | 2'-deoxycytidine | x | - | x |
| M21131 | dihydrocholesterol | x | - | x |
| M22030 | 2-hydroxyisobutyrate | x | - | x |
| M22116 | 4-methyl-2-oxopentanoate | x | - | x |
| M22189 | palmitoylcarnitine | x | - | x |
| M27414 | beta-sitosterol | x | - | x |
| M27672 | 3-indoxyl sulfate | x | - | x |
| M27710 | N-acetylglycine | x | - | x |
| M32492 | caprylate (8:0) | x | - | x |
| M32593 | heme*\|heme | x | - | x |
| M33230 | 1-palmitoleoylglycerophosphocholine* | x | - | x |
| M33422 | gamma-glutamylphenylalanine | x | - | x |
| M33441 | isobutyrylcarnitine | x | - | x |
| M33442 | pseudouridine | x | - | x |
| M33515 | X - 12100\|X - 12100\|hydroxytryptophan* | x | - | x |
| M33821 | 1-eicosatrienoylglycerophosphocholine* | x | - | x |
| M33945 | phenylacetylglycine | x | - | x |
| M33970 | cis-vaccenate (18:1n7) | x | - | x |
| M33997 (P) / M39511 (K) | campesterol | x | - | x |
| M34258 | 2-docosahexaenoylglycerophosphoethanolamine* | x | - | x |
| M34407 | isovalerylcarnitine | x | - | x |
| M34409 | stearoylcarnitine | x | - | x |
| M34456 | gamma-glutamylisoleucine* | x | - | x |
| M34585 | 4-hydroxybutyrate (GHB) | x | - | x |
| M35107 | isovalerylglycine | x | - | x |
| M35127 | pro-hydroxy-pro | x | - | x |
| M35160 | oleoylcarnitine | x | - | x |
| M35253 | 2-palmitoylglycerophosphocholine* | x | - | x |
| M35254 | 2-oleoylglycerophosphocholine* | x | - | x |
| M35255 | 2-stearoylglycerophosphocholine* | x | - | x |
| M35431 | 2-methylbutyroylcarnitine | x | - | x |
| M35436 | hexanoylglycine | x | - | x |
| M35490 | Isobar: 2-propylpentanoic acid, 2-ethylhexanoic acid | x | - | x |
| M35623 | 1-arachidoylglycerophosphocholine | x | - | x |
| M35678 | hexadecanedioate | x | - | x |
| M35883 | 2-docosahexaenoylglycerophosphocholine* | x | - | x |
| M36747 | deoxycarnitine | x | - | x |
| M37366 | 2-docosapentaenoylglycerophosphocholine* | x | - | x |
| M16821 | X - 04498 | x | - | x |
| M19363 | X - 06227 | x | - | x |
| M21630 | X - 08402 | x | - | x |
| M22320 | X - 08889 | x | - | x |
| M32958 | X - 11641 | x | - | x |
| M33516 | X - 12101 | x | - | x |
| M33813 | X - 12385 | x | - | x |
| M34040 | X - 12510 | x | - | x |
| M34539 | X - 12855 | x | - | x |
| M35338 | X - 13560 | x | - | x |
| M36860 | X - 14839 | x | - | x |
| M37548 | X - 15301 | x | - | x |
| M39130 | X - 16557 | x | - | x |
| M39568 | X - 16982 | x | - | x |
| M39815 | X - 17151 | x | - | x |
| n = 118 |  |  |  |  |
| M01587 | N-acetylleucine | x | - | - |
| M01644 | heptanoate (7:0) | x | - | - |
| M01669 | 4-hydroxyphenylpyruvate | x | - | - |
| M01708 | 7,8-dihydrofolate | x | - | - |
| M05983 | corticosterone | x | - | - |
| M15129 | alanylalanine | x | - | - |
| M15573 | trehalose | x | - | - |
| M15778 | benzoate | x | - | - |
| M16822 | X - 04499\|X - 04499\|3,4-dihydroxybutyrate | x | - | - |
| M18349 | indolelactate | x | - | - |
| M21025 | iminodiacetate (IDA) | x | - | - |
| M21047 | 3-methyl-2-oxobutyrate | x | - | - |
| M22001 | 3-hydroxyoctanoate | x | - | - |
| M22132 | alpha-hydroxyisocaproate | x | - | - |
| M22177 | levulinate (4-oxovalerate) | x | - | - |
| M27738 | threonate | x | - | - |
| M32412 | butyrylcarnitine | x | - | - |
| M32458 | oleamide | x | - | - |
| M32511 | EDTA | x | - | - |
| M32910 | X - 11593\|O-methylascorbate\|X - 11593\|O-methylascorbate* | x | - | - |
| M33453 | alpha-ketoglutarate | x | - | - |
| M33871 | 1-eicosadienoylglycerophosphocholine* | x | - | - |
| M33937 | alpha-hydroxyisovalerate | x | - | - |
| M33946 | N-acetylhistidine | x | - | - |
| M33950 | N-acetylphenylalanine | x | - | - |
| M33957 | 1-heptadecanoylglycerophosphocholine | x | - | - |
| M33959 | N-acetyltryptophan | x | - | - |
| M33967 | N-acetylisoleucine | x | - | - |
| M34301 | TDTEDKGEFLSEGGGVR* | x | - | - |
| M35129 | dihydrobiopterin | x | - | - |
| M35174 | mead acid (20:3n9) | x | - | - |
| M35626 | 1-myristoylglycerophosphocholine | x | - | - |
| M35819 | 2-palmitoleoylglycerophosphocholine* | x | - | - |
| M36746 | 2-hydroxy-3-methylvalerate | x | - | - |
| M36754 | octadecanedioate | x | - | - |
| M36776 | 7-alpha-hydroxy-3-oxo-4-cholestenoate (7-Hoca) | x | - | - |
| M37063 | gamma-glutamylalanine | x | - | - |
| M37231 | 1-docosapentaenoylglycerophosphocholine* | x | - | - |
| M37432 | N-acetyl-beta-alanine | x | - | - |
| M37487 | X - 13183\|stearamide | x | - | - |
| M37536 | X - 12441\|12-HETE\|12-hydroxyeicosatetraenoate (12-HETE) | x | - | - |
| M37539 | gamma-glutamylmethionine | x | - | - |
| M38002 | 1,2-propanediol | x | - | - |
| M38293 | 13-methylmyristic acid | x | - | - |
| M38296 | 17-methylstearate | x | - | - |
| M38768 | 15-methylpalmitate (isobar with 2-methylpalmitate)\|X - 11319\|methyl palmitate (15 or 2) | x | - | - |
| M39831 | eicosanodioate | x | - | - |
| M12593 | X - 02973 | x | - | - |
| M12625 | X - 03002 | x | - | - |
| M12626 | X - 03003 | x | - | - |
| M12770 | X - 03090 | x | - | - |
| M16818 | X - 04495 | x | - | - |
| M17325 | X - 04766 | x | - | - |
| M18929 | X - 05907 | x | - | - |
| M19367 | X - 06266 | x | - | - |
| M19412 | X - 06349 | x | - | - |
| M19462 | X - 06446 | x | - | - |
| M22602 | X - 09045 | x | - | - |
| M24469 | X - 10266 | x | - | - |
| M25596 | X - 10426 | x | - | - |
| M27888 | X - 10609 | x | - | - |
| M31618 | X - 10964 | x | - | - |
| M32621 | X - 11304 | x | - | - |
| M32625 | X - 11308 | x | - | - |
| M32689 | X - 11372 | x | - | - |
| M32724 | X - 11407 | x | - | - |
| M32847 | X - 11530 | x | - | - |
| M32867 | X - 11550 | x | - | - |
| M33132 | X - 11787 | x | - | - |
| M33206 | X - 11861 | x | - | - |
| M33269 | X - 11662 | x | - | - |
| M33380 | X - 12029 | x | - | - |
| M33389 | X - 12038 | x | - | - |
| M33539 | X - 12124 | x | - | - |
| M33793 | X - 12365 | x | - | - |
| M34031 | X - 12505 | x | - | - |
| M34041 | X - 12511 | x | - | - |
| M34100 | X - 12537 | x | - | - |
| M34244 | X - 12644 | x | - | - |
| M34252 | X - 12649 | x | - | - |
| M34290 | X - 12681 | x | - | - |
| M34452 | X - 12775 | x | - | - |
| M34453 | X - 12776 | x | - | - |
| M34469 | X - 12786 | x | - | - |
| M34671 | X - 12987 | x | - | - |
| M34765 | X - 13073 | x | - | - |
| M35247 | X - 13483 | x | - | - |
| M35359 | X - 13581 | x | - | - |
| M36576 | X - 14649 | x | - | - |
| M36877 | X - 14850 | x | - | - |
| M37550 | X - 15303 | x | - | - |
| M37943 | X - 15694 | x | - | - |
| M38063 | X - 15808 | x | - | - |
| M38441 | X - 16000 | x | - | - |
| M38514 | X - 16071 | x | - | - |
| M38532 | X - 16089 | x | - | - |
| M38772 | X - 16206 | x | - | - |
| M38840 | X - 16271 | x | - | - |
| M39053 | X - 16480 | x | - | - |
| M39081 | X - 16508 | x | - | - |
| M39135 | X - 16562 | x | - | - |
| M39142 | X - 16569 | x | - | - |
| M39148 | X - 16575 | x | - | - |
| M39149 | X - 16576 | x | - | - |
| M39150 | X - 16577 | x | - | - |
| M39161 | X - 16588 | x | - | - |
| M39162 | X - 16589 | x | - | - |
| M39166 | X - 16593 | x | - | - |
| M39170 | X - 16597 | x | - | - |
| M39367 | X - 16786 | x | - | - |
| M39526 | X - 16943 | x | - | - |
| M39748 | X - 17111 | x | - | - |
| M39824 | X - 17160 | x | - | - |
| M39965 | X - 17257 | x | - | - |
| M39967 | X - 17259 | x | - | - |
| M39979 | X - 17269 | x | - | - |
| M40208 | X - 17383 | x | - | - |
| M40250 | X - 17425 | x | - | - |
| n = 119 |  |  |  |  |
| M00561 | ribose 5-phosphate | - | x | - |
| M01118 | arachidate (20:0) | - | x | - |
| M01361 | pentadecanoate (15:0) | - | x | - |
| M01516 | sarcosine (N-Methylglycine) | - | x | - |
| M01566 | 3-aminoisobutyrate | - | x | - |
| M01605 | ursodeoxycholate | - | x | - |
| M02127 | glutathione, reduced (GSH) | - | x | - |
| M02936 | coenzyme A | - | x | - |
| M12261 | taurodeoxycholate | - | x | - |
| M12358 | biopterin | - | x | - |
| M15095 | N-acetylglucosamine | - | x | - |
| M15499 | betaine aldehyde | - | x | - |
| M15504 | phosphopantetheine | - | x | - |
| M15691 | adenosine 3',5'-diphosphate | - | x | - |
| M15806 | maltose | - | x | - |
| M15860 | UDP-galactose | - | x | - |
| M15877 | maltotriose | - | x | - |
| M15910 | maltotetraose | - | x | - |
| M15915 | S-adenosylmethionine (SAM) | - | x | - |
| M16829 | pyroglutamine* | - | x | - |
| M17987 | X - 05228 | - | x | - |
| M18289 | 3'-dephosphocoenzyme A | - | x | - |
| M18319 | 5-aminovalerate | - | x | - |
| M18330 | 5-methyltetrahydrofolate (5MeTHF) | - | x | - |
| M18494 | taurochenodeoxycholate | - | x | - |
| M22842 | cholate | - | x | - |
| M37256 | glucose-6-phosphate (G6P) | - | x | - |
| M31885 | beta-muricholate | - | x | - |
| M32417 | docosatrienoate (22:3n3) | - | x | - |
| M33942 | N-acetylasparagine | - | x | - |
| M33943 | N-acetylglutamine | - | x | - |
| M33944 | S-methylglutathione | - | x | - |
| M34418 | cytidine 5'-diphosphocholine | - | x | - |
| M34437 | 1-stearoylglycerophosphoglycerol | - | x | - |
| M35142 | adenosine 3'-monophosphate (3'-AMP) | - | x | - |
| M35163 | maltopentaose | - | x | - |
| M35170 | maltohexaose | - | x | - |
| M35676 | dihomo-gamma-linolenate (20:3n6) | - | x | - |
| M35717 | phosphoglycerate (2 or 3) | - | x | - |
| M35855 | ribulose | - | x | - |
| M35864 | tagatose | - | x | - |
| M36602 | 1-oleoylglycerophosphoinositol* | - | x | - |
| M36752 | N6-acetyllysine | - | x | - |
| M36815 | adenosine 2'-monophosphate (2'-AMP) | - | x | - |
| M36857 | succinyl CoA | - | x | - |
| M37059 | malonylcarnitine | - | x | - |
| M37076 | N-acetylserine | - | x | - |
| M37288 | Isobar: ribulose 5-phosphate, xylulose 5-phosphate | - | x | - |
| M38399 | 9,10-hydroxyoctadec-12(Z)-enoic acid | - | x | - |
| M39378 | tauroursodeoxycholate | - | x | - |
| M39541 | thiamin (Vitamin B1) | - | x | - |
| M39609 | 16-hydroxypalmitate | - | x | - |
| M40266 | homocysteine | - | x | - |
| M37372 | 5-HETE | - | x | - |
| M39732 | oleoyltaurine | - | x | - |
| M19961 | X - 06913 | - | x | - |
| M25436 | X - 10356 | - | x | - |
| M26418 | X - 10460 | - | x | - |
| M27272 | X - 10505 | - | x | - |
| M28774 | X - 10677 | - | x | - |
| M32897 | X - 11580 | - | x | - |
| M32900 | X - 11583 | - | x | - |
| M32901 | X - 11584 | - | x | - |
| M32908 | X - 11591 | - | x | - |
| M32909 | X - 11592 | - | x | - |
| M32915 | X - 11598 | - | x | - |
| M39413 | X - 11611 | - | x | - |
| M32934 | X - 11617 | - | x | - |
| M32936 | X - 11619 | - | x | - |
| M32944 | X - 11627 | - | x | - |
| M32946 | X - 11629 | - | x | - |
| M32947 | X - 11630 | - | x | - |
| M32948 | X - 11631 | - | x | - |
| M32955 | X - 10458 | - | x | - |
| M32966 | X - 02029 | - | x | - |
| M33026 | X - 11684 | - | x | - |
| M33060 | X - 11715 | - | x | - |
| M33066 | X - 10593 | - | x | - |
| M33069 | X - 11724 | - | x | - |
| M33106 | X - 11761 | - | x | - |
| M33259 | X - 11914 | - | x | - |
| M33346 | X - 11639 | - | x | - |
| M33360 | X - 12014 | - | x | - |
| M33907 | X - 12462 | - | x | - |
| M34062 | X - 12524 | - | x | - |
| M34224 | X - 12630 | - | x | - |
| M34266 | X - 12661 | - | x | - |
| M34270 | X - 12665 | - | x | - |
| M34483 | X - 12800 | - | x | - |
| M34580 | X - 12896 | - | x | - |
| M34637 | X - 12953 | - | x | - |
| M34670 | X - 12986 | - | x | - |
| M34676 | X - 12992 | - | x | - |
| M34776 | X - 13084 | - | x | - |
| M34912 | X - 13215 | - | x | - |
| M34927 | X - 13230 | - | x | - |
| M35140 | X - 13414 | - | x | - |
| M35215 | X - 13452 | - | x | - |
| M35262 | X - 13489 | - | x | - |
| M35276 | X - 13502 | - | x | - |
| M35314 | X - 13537 | - | x | - |
| M35582 | X - 13772 | - | x | - |
| M36521 | X - 14594 | - | x | - |
| M36552 | X - 14625 | - | x | - |
| M36556 | X - 14629 | - | x | - |
| M36965 | X - 14938 | - | x | - |
| M37794 | X - 15545 | - | x | - |
| M37795 | X - 15546 | - | x | - |
| M37804 | X - 15555 | - | x | - |
| M38370 | X - 15937 | - | x | - |
| M39386 | X - 16805 | - | x | - |
| M39554 | X - 16968 | - | x | - |
| M39633 | X - 17002 | - | x | - |
| M39639 | X - 17008 | - | x | - |
| M39649 | X - 17018 | - | x | - |
| M39652 | X - 17021 | - | x | - |
| M39660 | X - 17029 | - | x | - |
| M39819 | X - 17155 | - | x | - |
| M40234 | X - 17409 | - | x | - |
| n = 132 |  |  |  |  |
| M00531 | 3-hydroxy-3-methylglutarate | - | - | x |
| M00533 | 2'-deoxycytidine 5'-monophosphate | - | - | x |
| M00604 | thymine | - | - | x |
| M01411 | 2'-deoxyguanosine | - | - | x |
| M01412 | 2'-deoxyuridine | - | - | x |
| M01417 | kynurenate | - | - | x |
| M01559 | 5,6-dihydrouracil | - | - | x |
| M01591 | N-acetylvaline | - | - | x |
| M02372 | cytidine 5'-monophosphate (5'-CMP) | - | - | x |
| M15488 | acetylphosphate | - | - | x |
| M15747 | anserine | - | - | x |
| M15753 | hippurate | - | - | x |
| M17747 | sphingosine | - | - | x |
| M17769 | sphinganine | - | - | x |
| M18344 | xylulose | - | - | x |
| M18357 | glycylvaline | - | - | x |
| M20476 | 6-keto prostaglandin F1alpha | - | - | x |
| M21030 | glycylglycine | - | - | x |
| M21188 | 1-stearoylglycerol (1-monostearin) | - | - | x |
| M22053 | 3-hydroxydecanoate | - | - | x |
| M22158 | citramalate | - | - | x |
| M22175 | aspartylphenylalanine | - | - | x |
| M23642 | homoserine | - | - | x |
| M31928 | 2-methylbutyrylglycine | - | - | x |
| M31940 | 3-methylcrotonylglycine | - | - | x |
| M32379 | scyllo-inositol | - | - | x |
| M32740 | X - 11423\|O-sulfo-L-tyrosine | - | - | x |
| M33419 | 2-palmitoylglycerol (2-monopalmitin) | - | - | x |
| M33468 | felinine* | - | - | x |
| M33487 | glutamate, gamma-methyl ester | - | - | x |
| M33939 | N-acetylthreonine | - | - | x |
| M34188 | 7-ketocholesterol | - | - | x |
| M34875 | 2-docosapentaenoylglycerophosphoethanolamine* | - | - | x |
| M35133 | N2-methylguanosine | - | - | x |
| M35135 | N3-methyluridine | - | - | x |
| M35137 | N2,N2-dimethylguanosine | - | - | x |
| M35629 | 2-phosphoglycerate | - | - | x |
| M35669 | tetradecanedioate | - | - | x |
| M35884 | 2-eicosatrienoylglycerophosphocholine* | - | - | x |
| M36808 | dimethylarginine (SDMA + ADMA) | - | - | x |
| M37058 | succinylcarnitine | - | - | x |
| M37070 | methylphosphate | - | - | x |
| M39270 | 1-palmitoylplasmenylethanolamine* | - | - | x |
| M39592 | S-methylcysteine | - | - | x |
| M40068 | aspartylleucine | - | - | x |
| M17388 | X - 04795 | - | - | x |
| M19959 | X - 06911 | - | - | x |
| M21390 | X - 08166 | - | - | x |
| M21393 | X - 08168 | - | - | x |
| M21397 | X - 08172 | - | - | x |
| M22052 | X - 08791 | - | - | x |
| M22308 | X - 08886 | - | - | x |
| M22344 | X - 08895 | - | - | x |
| M22480 | X - 08987 | - | - | x |
| M27278 | X - 10510 | - | - | x |
| M29315 | X - 10680 | - | - | x |
| M32057 | X - 11081 | - | - | x |
| M32650 | X - 11333 | - | - | x |
| M32651 | X - 11334 | - | - | x |
| M32653 | X - 03249 | - | - | x |
| M32854 | X - 11537 | - | - | x |
| M32898 | X - 11581 | - | - | x |
| M32935 | X - 11618 | - | - | x |
| M33028 | X - 01497 | - | - | x |
| M33330 | X - 11984 | - | - | x |
| M33379 | X - 12028 | - | - | x |
| M33525 | X - 12110 | - | - | x |
| M33529 | X - 12114 | - | - | x |
| M33530 | X - 12115 | - | - | x |
| M33534 | X - 12119 | - | - | x |
| M33542 | X - 12127 | - | - | x |
| M33543 | X - 12128 | - | - | x |
| M33625 | X - 12156 | - | - | x |
| M33760 | X - 12335 | - | - | x |
| M33764 | X - 12339 | - | - | x |
| M34170 | X - 12602 | - | - | x |
| M34479 | X - 12796 | - | - | x |
| M34502 | X - 12819 | - | - | x |
| M34556 | X - 12872 | - | - | x |
| M34583 | X - 12899 | - | - | x |
| M34675 | X - 12991 | - | - | x |
| M34681 | X - 12997 | - | - | x |
| M34691 | X - 13006 | - | - | x |
| M35087 | X - 13387 | - | - | x |
| M35091 | X - 13391 | - | - | x |
| M35124 | X - 13409 | - | - | x |
| M35134 | X - 13411 | - | - | x |
| M35148 | X - 13418 | - | - | x |
| M35232 | X - 13469 | - | - | x |
| M35286 | X - 13512 | - | - | x |
| M35323 | X - 13545 | - | - | x |
| M35553 | X - 13743 | - | - | x |
| M35760 | X - 13865 | - | - | x |
| M36011 | X - 14088 | - | - | x |
| M36073 | X - 14147 | - | - | x |
| M36310 | X - 14384 | - | - | x |
| M37265 | X - 15124 | - | - | x |
| M37302 | X - 15161 | - | - | x |
| M37305 | X - 15151 | - | - | x |
| M37310 | X - 15169 | - | - | x |
| M37362 | X - 15220 | - | - | x |
| M37564 | X - 15317 | - | - | x |
| M37594 | X - 15347 | - | - | x |
| M37709 | X - 15461 | - | - | x |
| M37716 | X - 15468 | - | - | x |
| M37720 | X - 15472 | - | - | x |
| M37745 | X - 15497 | - | - | x |
| M37757 | X - 15509 | - | - | x |
| M37761 | X - 15513 | - | - | x |
| M37764 | X - 15516 | - | - | x |
| M37768 | X - 15520 | - | - | x |
| M37769 | X - 15521 | - | - | x |
| M37775 | X - 15523 | - | - | x |
| M37778 | X - 15530 | - | - | x |
| M37780 | X - 15532 | - | - | x |
| M37854 | X - 15605 | - | - | x |
| M37874 | X - 15625 | - | - | x |
| M37915 | X - 15666 | - | - | x |
| M38508 | X - 16065 | - | - | x |
| M38537 | X - 16094 | - | - | x |
| M38815 | X - 16247 | - | - | x |
| M38819 | X - 16250 | - | - | x |
| M38821 | X - 16252 | - | - | x |
| M39015 | X - 16444 | - | - | x |
| M39146 | X - 16573 | - | - | x |
| M39179 | X - 16606 | - | - | x |
| M39199 | X - 16626 | - | - | x |
| M39200 | X - 16627 | - | - | x |
| M39558 | X - 16972 | - | - | x |
| M39572 | X - 16986 | - | - | x |
| M39642 | X - 17011 | - | - | x |
| M39653 | X - 17022 | - | - | x |

**Supplementary Table 2.** **Metformin-associated metabolites in murine plasma, liver and kidney**

Regression results of beta-estimate (β) with 95% confidence interval (CI) per standard deviation (s.d.) and *P*-value of two pairwise comparisons in murine plasma are shown. Metformin-treated diabetic mice (MET, n = 10), vehicle-gavaged diabetic mice (VG, n = 10) and wild type mice (WT, n = 10). *P*-values shown in bold indicates metabolites with Bonferroni correction (*P* < 0.05 / 351 = 1.42x10^-04^ for plasma, *P* < 0.05 / 391 = 1.28x10^-4^ for liver, *P*< 0.05 / 447 = 1.11x10^-04^ for kidney).

| **Metabolon ID** | **Metabolite name** | **MET vs. VG** | | **VG vs. WT** | | |
| --- | --- | --- | --- | --- | --- | --- |
|  |  | **β (95% CI) per s.d.** | ***P*-value** | **β (95% CI) per s.d.** | ***P*-value** | |
| **Plasma** | | | | | | |
| M01643 | fumarate | 0.82 (0.59, 1.06) | **6.73x10^-07^** | 0.3 (0.02, 0.58) | | 0.035 |
| M01303 | malate | 0.68 (0.42, 0.94) | **3.31x10^-05^** | 0.5 (0.18, 0.81) | | 4.11x10^-03^ |
| M33453 | alpha-ketoglutarate (α-KG) | 0.87 (0.59, 1.16) | **4.15x10^-06^** | 0.26 (-0.04, 0.56) | | 0.091 |
| M34585 | 4-hydroxybutyrate (4-HB) | 0.47 (0.29, 0.65) | **3.78x10^-05^** | -0.57 (-0.86, -0.27) | | 7.99x10^-04^ |
| M00605 | uracil | 0.58 (0.34, 0.82) | **8.39x10^-05^** | -0.27 (-0.44, -0.10) | | 4.32x10^-03^ |
| M02132 | citrulline | -0.89 (-1.12, -0.65) | **2.56x10^-07^** | 0.2 (-0.01, 0.41) | | 0.061 |
| M32348 | 2-aminobutyrate (2-AB) | 0.48 (0.28, 0.68) | **8.58x10^-05^** | -0.08 (-0.37, 0.20) | | 0.053 |
| **Liver** | | | | | | |
| M00057 | glutamate | 0.47 (0.31, 0.63) | **7.19x10^-06^** | -0.23 (-0.43, -0.02) | | 0.033 |
| M32975 | taurine | -0.14 (-0.19, -0.08) | **1.27x10^-04^** | -0.03 (-0.08, 0.01) | | 0.155 |
| M01643 | fumarate | 0.45 (0.17, 0.74) | 3.65x10^-03^ | -0.47 (-0.75, -0.19) | | 2.14x10^-03^ |
| M01303 | malate | 0.39 (0.16, 0.61) | 1.78x10^-03^ | -0.43 (-0.67, -0.18) | | 1.94x10^-03^ |
| **Kidney** | | | | | | |
| M37253 | 2-hydroxyglutarate (2-HG) | 0.66 (0.42, 0.90) | **2.06x10^-05^** | -0.65 (-0.91, -0.39) | | **4.84x10^-05^** |
| M01577 | 2-aminobutyrate (2-AB) | 0.57 (0.35, 0.80) | **5.06x10^-05^** | -0.23 (-0.50, 0.04) | | 0.093 |
| M01559 | 5,6-dihydrouracil | 0.39 (0.23, 0.55) | **6.86x10^-05^** | -0.04 (-0.29, 0.21) | | 0.737 |
| M01303 | malate | 0.14 (0.02, 0.27) | 0.022 | 0.29 (0.12, 0.45) | | 1.70x10^-03^ |

**Supplementary Table 3.** **Corroboration of metformin-associated metabolites in human studies**

Regression results of Beta-estimate (β) with 95% confidence interval (CI) per standard deviation (s.d.) and *P*‑value for each metabolite in the pairwise comparisons between metformin-treated T2D (mt‑T2D) patients with non-anti-diabetic drug-treated type 2 diabetes (ndt‑T2D) ones. Linear regression analysis adjusted for age, sex, body mass index, physical activity, high alcohol intake, smoking status, systolic blood pressure, HbA_1C_, fasting glucose, high density lipoprotein cholesterol, triglycerides. Significant metabolites are highlighted in bold (*P*< 0.05 / 3 = 1.67 x 10^-2^).

| **Metabolite** | **KORA F4** | | **QBB** | |  |
| --- | --- | --- | --- | --- | --- |
|  | **β (95% CI) per s.d.** | ***P*-value** | **β (95% CI) per s.d.** | ***P*-value** |  |
|  | 70 mt-T2D vs. 114 ndt-T2D | | 146 mt-T2D vs. 148 ndt-T2D | |  |
| Malate | 0.52 (0.18, 0.86) | **3.13 x 10^-03^** | 0.68 (0.36, 1.01) | **4.53 x 10^-05^** |  |
| Citrulline | -0.79 (-1.15, -0.43) | **2.54 x 10^-05^** | -1.15 (-1.50, -0.80) | **3.33 x 10^-10^** |  |
| 2-aminobutyrate | -0.11 (-0.43, 0.21) | 0.497 | -0.07 (-0.32, 0.19) | 0.596 |  |
|  |  | |  | | |

**Supplementary Table 4**. **Validation of two metabolites in longitudinal KORA S4-F4 study**

Regression results of Beta-estimate (β) with 95% confidence interval (CI) per standard deviation (s.d.) and *P*‑value for malate and citrulline in basic and full models. **a**: 34 T2D patients that started metformin therapy after baseline (S4) examination and 628 metformin-naïve participants, **b**-**e**: age- and sex-matched case-control studies with ratios of 1:1 (**a**), 1:2 (**b**), 1:4 (**c**) and 1:10 (**e**). Generalized estimating equations (GEE) adjusted for basic model (age and sex) and full model (age, sex, body mass index, physical activity, high alcohol intake, smoking status, systolic blood pressure, HbA_1C_, fasting glucose, high density lipoprotein cholesterol, triglycerides). Nominal significant metabolites are highlighted in bold (*P*< 0.05).

| **Metabolite** | **Basic GEE Model** | | **Full GEE Model** | |
| --- | --- | --- | --- | --- |
|  | **β (95% CI) per s.d.** | ***P*-value** | **β (95% CI) per s.d.** | ***P*-value** |
| **a**: population-based study (34:628) | | | | |
| Malate | 0.39 (0.18, 0.60) | **3.31 x 10^-04^** | 0.25 (0.01, 0.49) | **0.044** |
| Citrulline | -0.67 (-0.98, -0.36) | **2.03 x 10^-05^** | -0.61 (-0.94, -0.28) | **2.96 x 10^-04^** |
| **b**: age- and sex-matched case-control study with a 1:1 ratio (34:34) | | | |  |
| Malate | 0.09 (0.03, 0.15) | **2.72 x 10^-03^** | 0.10 (0.03, 0.16) | **2.17 x 10^-03^** |
| Citrulline | -0.07 (-0.11, -0.02) | **3.64 x 10^-03^** | -0.06 (-0.11, -0.02) | **3.86 x 10^-03^** |
| **c**: age- and sex-matched case-control study with a 1:2 ratio (34:68) | | | | |
| Malate | 0.07 (0.02, 0.12) | **3.64 x 10^-03^** | 0.07 (0.02, 0.12) | **4.06 x 10^-03^** |
| Citrulline | -0.07 (-0.11, -0.03) | **9.81 x 10^-04^** | -0.07 (-0.11, -0.03) | **3.23 x 10^-04^** |
| **d**: age- and sex-matched case-control study with a 1:4 ratio (34:136) | | | | |
| Malate | 0.06 (0.02, 0.11) | **3.46 x 10^-03^** | 0.06 (0.02, 0.11) | **7.41 x 10^-03^** |
| Citrulline | -0.06 (-0.10, -0.03) | **7.58 x 10^-04^** | -0.07 (-0.10, -0.03) | **1.26 x 10^-04^** |
| **e**: age- and sex-matched case-control study with a 1:10 ratio (34:340) | | | | |
| Malate | 0.05 (0.02, 0.09) | **5.72 x 10^-03^** | 0.04 (0.00, 0.08) | **0.043** |
| Citrulline | -0.07 (-0.10, -0.03) | **1.77 x 10^-04^** | -0.07 (-0.11, -0.04) | **2.57 x 10^-05^** |

**Supplementary Table 5**. **Combination therapy altered metabolites in murine plasma, liver and kidney**

Regression results of beta-estimate (β) and *P*-value of pairwise comparison SGLT2i+MET with MET in murine plasma, liver and kidney are shown. Nominal significant metabolites are highlighted in bold (*P*< 0.05).

| **Metabolite** | **Plasma** | |  | **Liver** | |  | **Kidney** | |
| --- | --- | --- | --- | --- | --- | --- | --- | --- |
|  | **β (95% CI)** | ***P*-value** |  | **β (95% CI)** | ***P*-value** |  | **β (95% CI)** | ***P*-value** |
| butyrylglycine | -0.78 (0.54, 1.01) | **4.24 x 10^-06^** |  | 0.52 (0.24, 0.79) | **1.78 x 10^-03^** |  | 0.46 (0.13, 0.80) | **0.015** |
| N-acetylglycine | 1.00 (0.62, 1.38) | **6.60 x 10^-05^** |  | - | - |  | 0.25 (0.04, 0.46) | **0.035** |
| indolelactate | 0.79 (0.49, 1.09) | **6.95 x 10^-05^** |  | - | - |  | - | - |
| X-10460 | - | - |  | -1.71 (-2.34, -1.07) | **4.90 x 10^-05^** |  | - | - |
| choline | 0.26 (0.08, 0.43) | **9.32 x 10^-03^** |  | 0.23 (0.14, 0.31) | **4.01 x 10^-05^** |  | - | - |
| X-12465 | 0.95 (0.53, 1.37) | **2.92 x 10^-03^** |  | 0.76 (0.28, 1.23) | **5.91 x 10^-03^** |  | 0.83 (0.39, 1.27) | **1.65 x 10^-03^** |
| pyruvate | -0.33 (-0.50, -1.57) | **1.52 x 10^-03^** |  | - | - |  | 0.28 (-0.16, 0.72) | 0.224 |
| α-KG | -0.64 (-0.98, -0.30) | **1.56 x 10^-03^** |  | - | - |  | - | - |
| fumarate | -0.34 (-0.71, 0.02) | 0.079 |  | -0.23 (-0.45, -0.01) | 0.051 |  | 0.03 (-0.11, 0.16) | 0.713 |
| malate | -0.33 (-0.57, -0.08) | **0.019** |  | -0.20 (-0.36, -0.04) | **0.021** |  | 0.02 (-0.12, 0.12) | 0.977 |
| taurine | -0.02 (-0.34, 0.30) | 0.894 |  | 0.13 (0.07, 0.19) | **6.24 x 10^-04^** |  | -0.05 (-0.20, 0.10) | 0.497 |
| glutamate | - | - |  | -0.17 (-0.30, -0.04) | **0.022** |  | 0.02 (-0.06, 0.11) | 0.647 |
| citrulline | -0.04 (-0.25, 0.18) | 0.752 |  | 0.16 (0.06, 0.25) | **4.86 x 10^-03^** |  | - | - |
| 2-HG | 0.01 (-0.20, 0.17) | 0.896 |  | -0.15 (-0.33, 0.03) | 0.110 |  | -0.39 (-0.64, -0.13) | **7.45 x 10^-03^** |
|  |  |  |  |  |  |  |  |  |

Additional File 2

**Detailed description of the untargeted method:**

Serum metabolites from KORA participants and plasma metabolites from mice were quantified at Metabolon Inc (Durham, NC) using a non-targeted metabolomics approach using gas and liquid chromatography coupled to mass spectrometry (GC/MS and LC/MS, respectively). Details of the methods used to quantify and identify metabolites were reported previously [1–3].

Sample preparation was performed on a Hamilton MLStar robotic system (Hamilton Company, Salt Lake City, UT): After thawing, each 100 µl of serum sample in a 96-well plate format was added to 400 µl of extraction solvent (methanol containing recovery standards). A Geno/Grinder 2000 (Glen Mills Inc., Clifton, NJ) was used for extraction by shaking for 2 min. After centrifugation, the supernatants were divided into four aliquots: two for LC/MS analysis (positive and negative electrospray ionization [ESI] modes), one for GC/MS analysis, and one for reserve aliquot. Samples were dried overnight under a vacuum to remove the solvent using a TurboVap (Zymark). Reconstitution was performed with 0.1% formic acid for LC/MS positive ion mode and 6.5mM ammonium bicarbonate (pH 8.0) for negative ion mode. Internal standards were included in both reconstitution solvents. GC/MS aliquots were derivatized with N, O-bis trimethylsilyl-trifluoroacetamide in an acetonitrile/dichloromethane/cyclohexane (5:4:1) solvent mixture containing 5% triethylamine and retention time markers for 1 hour at 60°C.

LC/MS analysis was performed on an LTQ mass spectrometer (Thermo Fisher Scientific, Waltham, MA). The instrument was equipped with a Waters Acquity UPLC system (Waters Corporation, Milford, MA). Acidic (solvent A: 0.1% formic acid in H2O; solvent B: 0.1% formic acid in methanol) and basic (solvent A: 6.5 mM ammonium bicarbonate, pH 8.0; and solvent B: 6.5 mM ammonium bicarbonate in 98% methanol) mobile phase conditions optimized for positive and negative ESI, respectively, were used on two separate columns (2.1×100 mm, BEH C18 1.7 µm particle; Waters Corporation). The columns were developed in a gradient from 100% A to 98% B over a run time of 11 minutes at a flow rate of 350 µl/min after injection of the sample extracts. The LTQ mass spectrometer ESI source was connected directly to the eluent flow. Full scan mass spectra (mass to charge ratio [m/z] of 99 - 1000) and data-dependent MS/MS scans with dynamic exclusion were recorded in alternation.

GC/MS analysis was performed on a Thermo-Finnigan Trace DSQ fast-scanning single-quadrupole mass spectrometer. The instrument was equipped with a 20 m × 0.18 mm GC column with a 0.18 µm film phase of 5% phenyldimethylsilicone. The column temperature was ramped between 60°C and 340°C using helium as the carrier gas and electron ionization at 70 eV. Mass spectra were recorded within the scan range of 50 to 750 m/z.

Metabolite identification was performed by semi-automated multi-parameter comparison of LC-MS and GC-MS data with a proprietary library containing retention time, m/z, and associated adduct/fragment spectra. Rigorous and consistent data cleaning procedures were applied separately to the two datasets to account for differences in sample collection times and because metabolite content in untargeted metabolomics experiments may vary somewhat over time for technical reasons. To correct for inter-day variation in measurements, the raw area counts for each metabolite were normalized to the median of the day of the run.

**References**

1. Evans AM, DeHaven CD, Barrett T, Mitchell M, Milgram E. Integrated, nontargeted ultrahigh performance liquid chromatography/electrospray ionization tandem mass spectrometry platform for the identification and relative quantification of the small-molecule complement of biological systems. Anal Chem. 2009;81:6656–67.
2. Dehaven CD, Evans AM, Dai H, Lawton KA. Organization of GC/MS and LC/MS metabolomics data into chemical libraries. J Cheminform. 2010;2:9.
3. Sekula P, Goek ON, Quaye L, Barrios C, Levey AS, Römisch-Margl W, et al. A Metabolome-Wide Association Study of Kidney Function and Disease in the General Population. J Am Soc Nephrol. 2016;27:1175–88.
